# Using Separation-of-Function Mutagenesis To Define the Full Spectrum of Activities Performed by the Est1 Telomerase Subunit *in Vivo*

**DOI:** 10.1534/genetics.117.300145

**Published:** 2017-11-29

**Authors:** Johnathan W. Lubin, Timothy M. Tucey, Victoria Lundblad

**Affiliations:** * Division of Biological Sciences, University of California, San Diego, La Jolla, California 92093-0130; † Salk Institute for Biological Studies, La Jolla, California 92037-1099

**Keywords:** Est1, telomerase, telomere, separation-of-function, mutagenesis

## Abstract

A leading objective in biology is to identify the complete set of activities that each gene performs *in vivo*. In this study, we have asked whether a genetic approach can provide an efficient means of achieving this goal, through the identification and analysis of a comprehensive set of separation-of-function (*sof*^−^) mutations in a gene. Toward this goal, we have subjected the *Saccharomyces cerevisiae EST1* gene, which encodes a regulatory subunit of telomerase, to intensive mutagenesis (with an average coverage of one mutation for every 4.5 residues), using strategies that eliminated those mutations that disrupted protein folding/stability. The resulting set of *sof*^−^ mutations defined four biochemically distinct activities for the Est1 telomerase protein: two temporally separable steps in telomerase holoenzyme assembly, a telomerase recruitment activity, and a fourth newly discovered regulatory function. Although biochemically distinct, impairment of each of these four different activities nevertheless conferred a common phenotype (critically short telomeres) comparable to that of an *est1*-∆ null strain. This highlights the limitations of gene deletions, even for nonessential genes; we suggest that employing a representative set of *sof*^−^ mutations for each gene in future high- and low-throughput investigations will provide deeper insights into how proteins interact inside the cell.

## Abstract (precis)

A leading objective in biology is to identify the complete set of activities performed by each gene. Identification of a comprehensive set of separation...

TELOMERES—the ends of linear chromosomes, which are composed of G-rich repeats bound by an array of telomere-specific proteins—are essential for high-fidelity maintenance of linear chromosomes. Severe telomere dysfunction has catastrophic consequences for genome organization, but even modest reductions in telomere length can also have a genome-destabilizing effect. As a result, in cells that depend on long-term proliferation, a carefully regulated mechanism ensures that telomeres are stably maintained at an average length (Hug and Lingner 2006). In most eukaryotic species, a key player in this process is the enzyme telomerase. This telomere-dedicated enzyme is responsible for adding telomeric G-rich repeats onto the ends of chromosomes, thereby providing a counterbalance against sequence loss that arises due to incomplete DNA replication or other DNA-processing activities (Schmidt and Cech 2015; Wu *et al.* 2017). Although telomerase has been a topic of intense investigation, a detailed mechanistic picture of how telomerase-mediated elongation is regulated at individual telomeres is incomplete. For example, yeast telomerase only elongates a small subset of telomeres in a cell cycle, with a preference for shorter telomeres (Teixeira *et al.* 2004), a bias that also extends to mammalian telomeres (Britt-Compton *et al.* 2009). However, the molecular mechanism that restricts telomerase to a particular subset of telomeres has not yet been elucidated. Telomere length is also dictated by the number of telomeric repeats that are added each time telomerase interacts with its substrate, but how this enzymatic step is regulated *in vivo* is still poorly understood.

This incomplete picture suggests that there may be as-yet-undiscovered mechanisms that are critical for telomerase regulation. As one approach toward addressing this, we are constructing a functional surface map of yeast telomerase, by identifying functionally important amino acids on the surface of each telomerase subunit that, when mutated, disrupt specific activities. To facilitate the identification of these separation-of-function (*sof*^−^) mutations, we have employed a strategy that relies on overexpression dominant negative (ODN) phenotypes (*i.e.*, disruption of function in a wild-type strain in response to an overexpressed mutant protein) as a rapid means of identifying the rare subclass of mutations that target a specific biochemical property without affecting protein stability. The rationale is based on the premise, first elucidated by Ira Herskowitz (Herskowitz 1987), that mutant proteins must be structurally intact in order compete with the endogenous wild-type protein. In contrast, the much larger class of mutations that encode unstable/unfolded proteins will be phenotypically silent (or greatly attenuated) in an ODN-based assay.

We first applied this strategy to the Est3 subunit of telomerase, both as a proof-of-principle experiment and with the goal of identifying novel Est3 regulatory activities, by analyzing ODN phenotypes of a systematic set of mutations introduced into 20% of the amino acids in the Est3 protein. Notably, those mutant Est3 proteins with strong ODN phenotypes demonstrated a remarkably similar secondary structure content and thermal stability when compared to the wild-type protein (Lubin *et al.* 2013). Once the Est3 structure was solved (Rao *et al.* 2014), this revealed that every residue identified in our genetics-driven ODN screen was located on the Est3 protein surface. This provided a striking validation of this methodology, and argued that ODN-directed mutagenesis is capable of selectively identifying mutations in functionally important amino acids on the surface of a protein, even in the absence of structural information.

In this study, we have applied this protocol to the second of the three telomerase protein subunits, by analyzing a large panel of missense mutations in *EST1* for ODN effects on telomere length maintenance. The *sof*^−^ mutations recovered from this ODN screen correspond to three biochemically distinct activities: (i) a dual Est3-binding site that involves regions in both the N- and C-terminal halves of the Est1 protein; (ii) an expanded surface that mediates the Est1-Cdc13 interaction; and (iii) a newly defined regulatory activity that is not required for telomerase biogenesis or recruitment. In parallel, we performed an extensive loss-of-function (LOF) screen, which identified a novel 60-amino acid RNA-binding domain (RBD) that was phenotypically silent in the ODN assay. This combined ODN plus LOF analysis has provided a representative set of *sof*^−^ alleles that define four discrete activities performed by Est1. This provides a set of genetic reagents for *EST1* that has the potential to uncover activity-specific genetic interactions in future low- and high-throughput analyses, which would be otherwise masked by a complete deletion of the *EST1* gene.

## Materials and Methods

### Genetic analysis

The full list of strains and plasmids used in this study are described in Supplemental Material, Tables S1 and S2 in File S1, respectively. Standard genetic and molecular methods were used to introduce plasmids into yeast, introduce missense mutations into the *EST1* gene, and assess synthetic lethality in the *yku80*-∆ strain, as previously described (Lendvay *et al.* 1996; Lee *et al.* 2008; Lubin *et al.* 2013). Telomere length was assessed from two independent single colonies that were propagated for ∼75 generations, following transformation into either a wild-type yeast strain (for ODN assays) or an *est1*-∆ strain freshly generated by shuffling off a covering plasmid (for LOF assays).

### Biochemical analysis

For all of the biochemical experiments described in this study, mutations were integrated into the genome in place of the wild-type gene, as previously described (Paschini *et al.* 2012; Tucey and Lundblad 2014). This eliminates the possibility that effects on immunoprecipitation (IP) efficiency were due to incomplete gene expression by plasmid-borne alleles (as a consequence of either variations in plasmid copy number and/or incomplete promoters). Strains expressing integrated copies of both the wild-type *EST1* gene and mutant *est1* *sof*^−^ alleles exhibited a healthy (*i.e.*, nonsenescent) growth phenotype; PCR analysis was used to confirm that each mutant allele was integrated without unanticipated genomic rearrangements. Subsequent isolates that had lost the wild-type *EST1* gene and retained only the mutant *est1*^−^ allele (following propagation on 5-FOA) were confirmed by molecular analysis (by sequencing across the integrated mutant allele), as well as phenotypic analysis (telomere length and/or senescence), for every constructed strain. Whole-cell extracts were prepared from two independent 250 ml cultures (OD 0.8–0.9) for each genotype and processed in parallel. Cells were pelleted, washed in TMG200 (10 mM Tris-HCl pH 8, 1 mM MgCl_2_, 5% glycerol, and 200 mM NaCl_2_) + protease inhibitors + 0.1% Tween20, and resuspended in 1 ml of the same buffer. Extracts were prepared by grinding this 1-ml suspension in a mortar in the presence of liquid N_2_ until the suspension formed a fine powder. Extracts were clarified by three 10 min spins at 4° at 25,000 × *g*, and supernatants were immediately subjected to IP by incubation with anti-Flag M2 affinity gel (Sigma [Sigma Chemical], St. Louis, MO), in TMG200 + protease inhibitors + 0.1% Tween20 for 2 hr at 4°, with gentle rocking. Beads were washed 3× in the same buffer, and eluted for 4 min at 95° with TMG200 + 0.1% Tween20 equilibrated with SDS loading buffer + 0.7% β-mercaptoethanol. Immunoprecipitated proteins were resolved on 6% (for detection of Est1, Est2, or Pop1) or 12% (for detection of Est3 or Sme1) SDS-PAGE and probed with anti-myc 2272 (Cell Signaling Technology) at 1:1000 or anti-Flag F7425 (Sigma) at 1:10,000 dilution, followed by anti-rabbit IgG HRP conjugate (Promega, Madison, WI) at 1:10,000, and subsequent enhanced chemiluminescence (ECL) detection using preflashed film. ECL was used rather than alternative options (such as Li-cor Odyssey) due to substantially less background and a significantly higher signal-to-noise ratio; we previously demonstrated that this protocol can detect as little as twofold differences with high reproducibility over a 10-fold detection range (Tucey and Lundblad 2013).

### Data availability

The authors state that all data necessary for confirming the conclusions presented in the article are represented fully within the article.

## Results

### ODN-based mutagenesis identifies 11 candidate *sof*^−^ mutations in EST1

**Figure 1:**
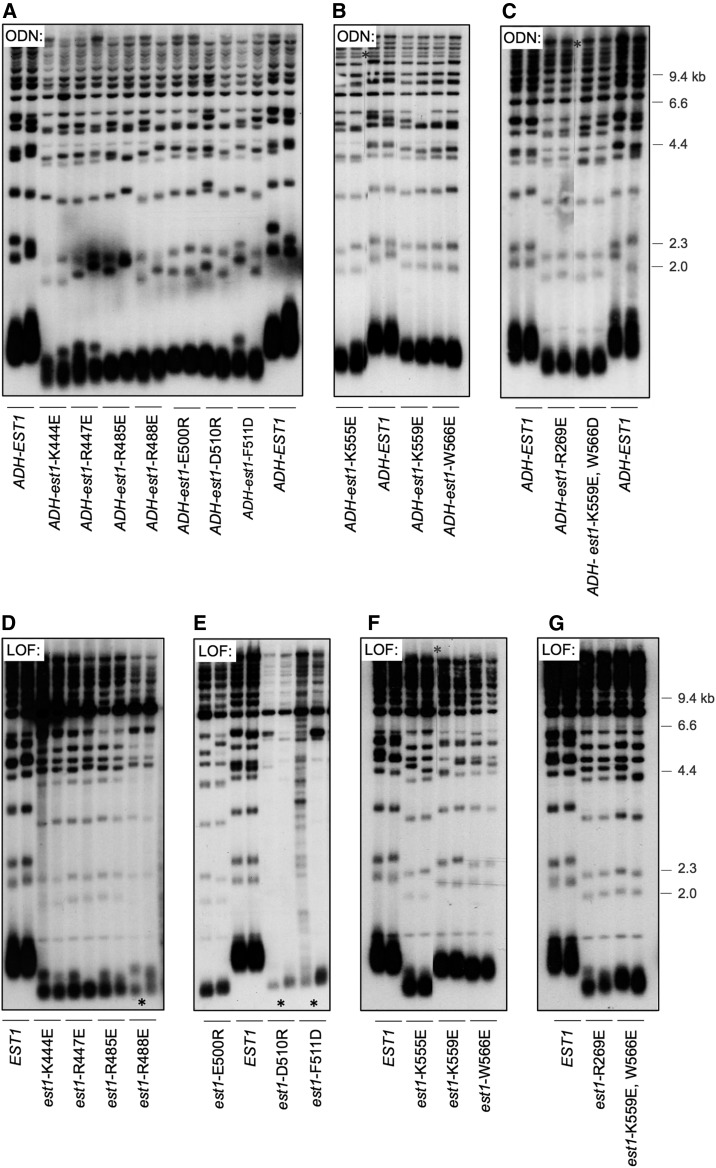
ODN mutagenesis identifies 11 candidate separation-of-function mutations in *EST1*. (A–C) Telomere length of wild yeast strains transformed with high-copy plasmids expressing either *EST1* or the indicated *est1*^−^ mutations, under control of the ADH promoter, assessed after ∼75 generations of growth. (D–G) Telomere length of *est1*-∆ strains transformed with single-copy plasmids with either *EST1* or the indicated *est1¯* mutations, expressed by the *EST1* promoter, determined after ∼75 generations of growth following transformation of the *est1*-∆ strain; the exception was three mutant strains (indicated by asterisks) that were examined at 25 generations (at this time point, these three strains were senescent and indistinguishable from an *est1*-∆ null strain). Since *est1*-K559E and *est1*-W566E conferred very modest *in vivo* phenotypes, these two mutations were combined to facilitate subsequent biochemical analysis. LOF, loss-of-function; ODN, overexpression dominant negative.

To identify and characterize functional surfaces on the Est1 protein, an ODN protocol was employed, whereby *est1*^−^ mutations were screened for the ability to disrupt telomere replication when overexpressed in the presence of the wild-type *EST1* gene. A total of 134 missense mutations were introduced by reverse mutagenesis into the *EST1* gene, which was present on a high-copy plasmid and under the control of the constitutive ADH promoter (Figure S1 in File S1). Amino acids were selected for mutagenesis based on sequence conservation (data not shown) and emphasized: (i) charged amino acids, based on our analysis indicating that mutation in residues in this category were more likely to encode a protein that retained structural stability (Lubin *et al.* 2013), and (ii) aromatic residues, which frequently mediate nucleic acid interactions (Jones *et al.* 2001; Baker and Grant 2007). This collection of 134 overexpressed *est1*^−^ mutations was transformed into a *yku80*-∆/p *CEN URA3 YKU80* strain, and transformants were screened for viability following loss of the *YKU80* plasmid, based on earlier observations showing that increased expression of mutant telomerase subunits confers inviability in a *yku80*-∆ strain (Lee *et al.* 2008; Lubin *et al.* 2013). This strategy identified 11 *est1*^−^ mutations that conferred a moderate to severe impact on viability when overexpressed in the *yku80*-∆ strain (Figure S2 in File S1), and also reduced telomere length when overexpressed in a wild-type (*i.e.*, *EST1 YKU80*) strain (Figure 1, A–C). There was a strong correlation between these two ODN phenotypes; for example, mutations with the most pronounced effects on viability in the *yku80*-∆ strain (Figure S2 in File S1) also conferred substantially shorter telomeres (Figure 1A), consistent with our prior observations with *EST3* (Lee *et al.* 2008; Lubin *et al.* 2013). These 11 *est1*^−^ mutations were subsequently assessed for effects on telomere length in a standard LOF assay, in which each allele, expressed by the *EST1* promoter on a single-copy plasmid, was transformed into an *est1*-∆ strain. Seven mutations conferred a severe impact on telomere length (Figure 1, D and E), whereas four mutations resulted in more intermediate phenotypes in the LOF assay (Figure 1, F and G). In each case, the severity of the ODN telomere length phenotype closely correlated with the strength of the corresponding LOF telomere length phenotype (compare Figure 1, A–C with Figure 1, D–G).

**Figure 2:**
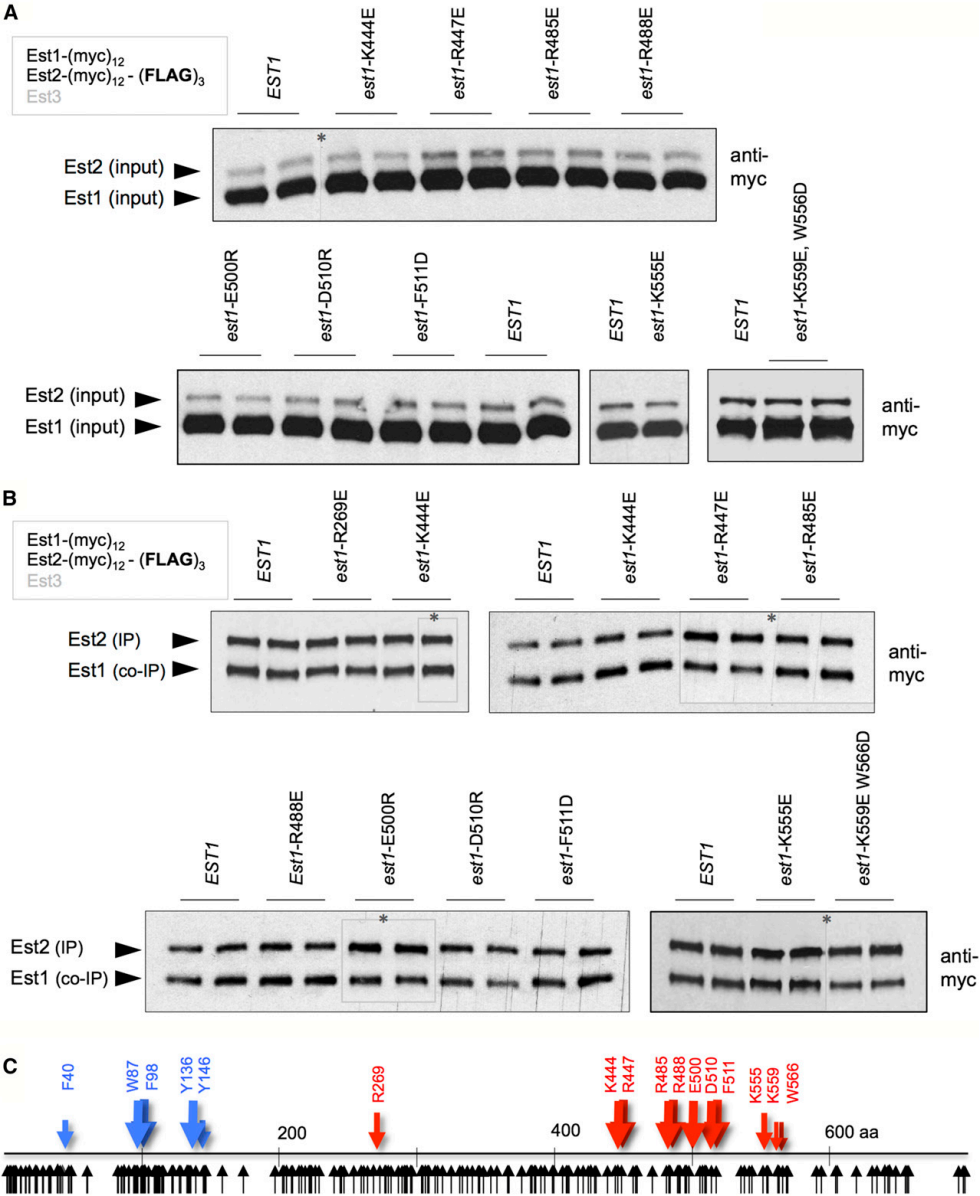
Formation of the Est1-TLC1-Est2 subcomplex is unimpaired by 11 separation-of-function mutations in *EST1*. (A and B) The relative levels of Est1 and Est2 proteins in extracts (A) and anti-Est2 IPs (B), as assessed by anti-myc westerns of anti-FLAG IPs prepared from strains with the indicated mutations, which were integrated into the genome in place of the wild-type *EST1* gene. Identical (myc)_12_ epitopes are present on the C- and N-termini of Est1 and Est2, respectively, with an additional (FLAG)_3_ epitope on Est2; different exposures were used (indicated by asterisks) in part (B), to ensure that the Est2 signal was the same for all of the images. (C) Schematic diagram of the Est1 protein, based on analysis shown in Figure 1 and Figure 3. The 11 *est1*^−^ mutations identified by ODN mutagenesis are indicated as red arrows, and the five RNA-binding-defective mutations, identified in Figure 3, as blue arrows. The black arrows below the line correspond to each mutation that was analyzed for ODN and/or LOF phenotypes; see Figure S1 in File S1 for a higher resolution image of the position of each mutagenized amino acid in the 699-amino acid Est1 protein. co-IP, coimmunoprecipitation; IPs, immunoprecipitations; LOF, loss-of-function; ODN, overexpression dominant negative.

We have previously argued that an ODN phenotype can distinguish between a mutation that encodes a structurally intact protein *vs.* a mutation that results in a nonspecific effect on protein stability/folding (Lubin *et al.* 2013). Consistent with this expectation, steady-state protein levels for each of these Est1 mutant proteins were comparable to that of the wild-type Est1 protein (Figure 2A). This was assessed in a strain bearing identical (myc)_12_ epitopes on Est1 and Est2, as well as a (FLAG)_3_ epitope on Est2, with each of the 11 *est1*^−^ mutations integrated into the genome. This allowed simultaneous detection of both proteins on the same anti-myc western, thereby providing highly accurate determination of the level of each mutant Est1 protein relative to the wild-type Est2 protein. As previously observed (Tucey and Lundblad 2013), the Est1 protein was present in at threefold excess, relative to Est2, in extracts prepared from asynchronous cultures; this ratio was unchanged in each of the mutant strains (Figure 2A). The Est1:Est2 ratio in the telomerase complex was also determined, following anti-FLAG IP of Est2. Examination of Est1 and Est2 protein levels in anti-FLAG IPs showed that each of the 11 mutant Est1 proteins was capable of forming a complex in a 1:1 ratio with Est2, in a manner that was indistinguishable from that of the wild-type Est1 protein (Figure 2B). Collectively, the observations shown in Figure 1 and Figure 2 argue that these mutations in *EST1* confer an *in vivo* defect without impairing protein stability and are candidates for *sof*^−^ mutations. The positions of these 11 mutations are indicated on a schematic diagram of the Est1 protein in Figure 2C.

### Identification of a novel RBD in Est1 that is conserved from yeast to humans

**Figure 3:**
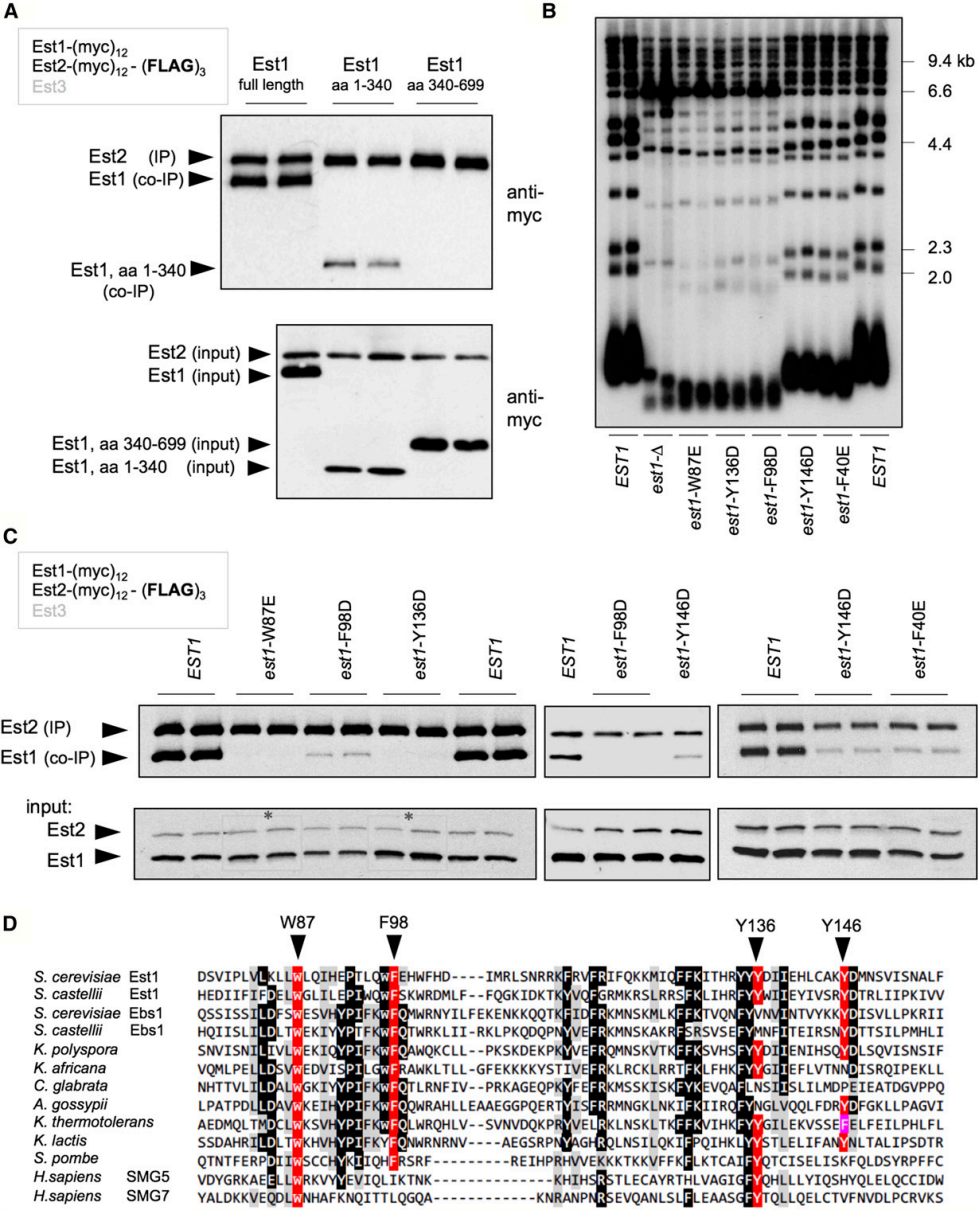
Identification of a novel 60-amino acid RNA-binding domain in Est1. (A) Co-IP of the N- (amino acids 1–340) or C-terminal (amino acids 340–699) domains of Est1 with Est2 was assessed by an anti-myc western of an anti-Est2 IP (top panel); no association between the C-terminal Est1 domain and Est2 was observed, even in a substantially darker exposure (Figure S4 in File S1), IP and extracts were resolved on 4–20% SDS-PAGE gradient gels. The reduced association of the N-terminal domain with Est2 compared to the full-length Est1 protein (top panel), was presumably due to reduced expression of this domain (bottom panel). (B) Telomere length of the five RNA-binding-defective *est1*^−^ mutant strains, determined after 75 generations of growth following introduction of single-copy plasmids with either *EST1* or the indicated mutations, expressed by the *EST1* promoter, into an *est1*-∆ strain. (C) The relative levels of Est1 and Est2 proteins in anti-Est2 IPs (top) and extracts (lower), assessed as in Figure 2. The slight increase in the levels of the Est1-Y136D protein in inputs, relative to Est2, was a reproducible observation; for the *est1*-W87E and *est1*-Y136D inputs, different exposures were used (as indicated by asterisks) to ensure that the Est2 signal was the same for each sample in this image. (D) The sequence of the RNA-binding domain from *S. cerevisiae* Est1 aligned with Est1 (or Ebs1) proteins from other yeasts, as well as the human SMG5 and SMG7 proteins; the alignment of the human proteins with yeast proteins is based on that in Fukuhara *et al.* (2005). co-IP, coimmunoprecipitation; IP, immunoprecipitation.

Notably, none of these 11 mutations affected the ability of Est1 to form the Est1-TLC1-Est2 preassembly complex (Tucey and Lundblad 2014), indicating that the ODN-based approach had failed to uncover mutations in the RNA-binding activity of Est1. As a first step toward identifying the region of Est1 responsible for RNA binding, the ability of two subdomains of Est1 to form a complex with TLC1 was examined. N- and C-terminal domains expressing amino acids 1–340 and 340–699, respectively, with an in-frame (myc)_12_ epitope, were integrated into the genome in place of the full-length Est1 protein, in a strain that also expressed the (FLAG)_3_-(myc)_12_-Est2 protein; domain boundaries were chosen based on a region of low sequence conservation around amino acids 340–350 (data not shown). Since TLC1 bridges the association between Est1 and Est2 through independent Est1-TLC1 and Est2-TLC1 interactions (Livengood *et al.* 2002; Lubin *et al.* 2012), the ability of an Est1 domain to co-IP with the Est2-TLC1 catalytic core reflects a direct interaction with TLC1. Following IP, the N-terminal domain of Est1 retained association with the Est2-TLC1 subcomplex, whereas the C-terminal domain was undetectable in anti-Est2 IPs (Figure 3A), indicating that RNA-binding activity localized to the N-terminal half of the Est1 protein.

Therefore, we subjected the N-terminal region of Est1 to conventional LOF analysis, by examining the telomere length of 84 *est1*^−^ mutations spanning amino acids 1–350 (Figures S1 and S3 in File S1). Each mutation, expressed on a single-copy plasmid by the *EST1* promoter, was introduced into an *est1*-∆ strain, and telomere length was examined after ∼75 generations of growth. This identified mutations in a cluster of five aromatic amino acids (from amino acids 40 to 146) that resulted in significant telomere shortening (Figure 3B; see also in Figure S3, A–N in File S1). To assess potential effects on RNA binding, these five mutations were introduced into the genome of the Est1-(myc)_12_ (FLAG)_3_-(myc)_12_-Est2 strain in place of the wild-type *EST1* gene, and the relative ratio of Est1 to Est2 in anti-Est2 immunoprecipitates was examined. We have previously shown that this assay provides a very sensitive read-out of the interaction between Est1 and TLC1, due to the ability to simultaneously detect Est1 and the Est2 subunit of the Est2-TLC1 catalytic core on the same anti-myc western; this assay has also been validated by our prior identification of a panel of mutations in TLC1 that disrupt Est1-TLC1 binding (Lubin *et al.* 2012; see also Figure S4 in File S1). Using this assay, all five mutant Est1 proteins exhibited a reduced ability to bind TLC1 to form an Est1-TLC1-Est2 subcomplex (Figure 3C). This was not simply a consequence of reduced Est1 protein levels, as shown by anti-myc westerns of extracts from these mutant strains (Figure 3C); in fact, the Est1-Y136D mutant protein reproducibly displayed a slight increase in protein levels, relative to wild-type Est1. There was also a striking correspondence between the degree of biochemical impairment and the *in vivo* consequences for telomere replication. Three mutations with a pronounced telomere length defect (*est1*-W87E, *est1*-F98D, and *est1*-Y136D) exhibited a substantial reduction in the interaction between Est1 and TLC1, whereas *est1*-F40 and *est1*-Y146D were only modestly diminished in both assays. When tested for ODN phenotypes, these five mutations failed to exhibit an ODN phenotype (Figure S4 in File S1 and data not shown), which explains our failure to identify this category of mutations in our ODN screen.

The recovery of five RNA-binding-defective *est1*^−^ mutations in residues with aromatic side chains, which are used extensively in RNA–protein interactions (Baker and Grant 2007), was not due to a mutation bias, as this region of Est1 was extensively mutagenized (Figure S1 in File S1). Furthermore, the region of Est1 that encompassed four of the residues identified by this analysis (Trp87, Phe98, Tyr136, and Tyr146) was highly conserved, as shown by the alignment that encompasses budding yeast, fission yeast, and human sequences (Figure 3D). These observations argue that we have uncovered a conserved, novel 60-amino acid RBD that does not exhibit sequence motifs characteristic of canonical RBDs (Helder *et al.* 2016). The position of the mutations that define the Est1 RBD are shown in Figure 2C. We note that the results presented here differ from observations from an earlier study, which reported that a different domain of the fission yeast Est1 protein was employed for RNA binding (Webb and Zakian 2012); Figure S4 in File S1 provides more information regarding the differences between these two studies.

### The Est1-Est3 interaction involves dual sites in the N- and C-terminal domains of Est1

**Figure 4:**
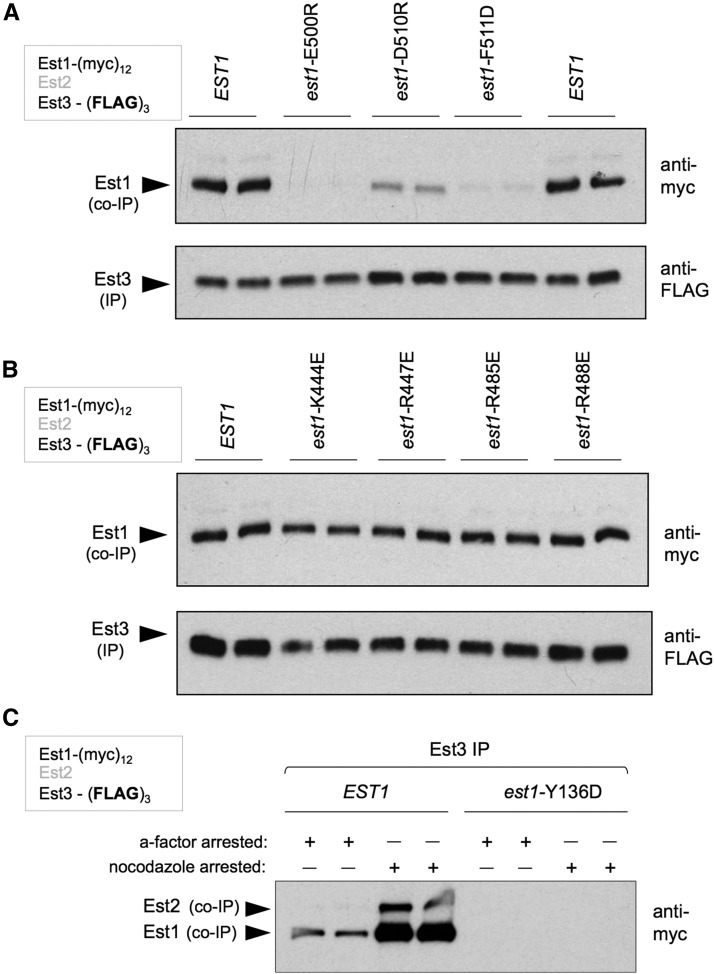
A cluster of residues in the C-terminal domain of Est1 mediate the Est1-Est3 interaction. (A and B) Association between Est1 and Est3, tagged with (myc)_12_ and (FLAG)_3_ epitopes, respectively, was monitored by anti-FLAG co-IP. A cluster of three mutations spanning an 11-amino acid region in the C-terminal region of Est1 substantially reduced the Est1-Est3 interaction (A), whereas other *sof*^−^ mutations in the C-terminal domain had no effect (B). (C) Est3 failed to associate with the RNA-binding-defective Est1-Y136D mutant protein, as assessed by Est1-Est3 co-IPs performed as in (A). co-IP, coimmunoprecipitation; IP, immunoprecipitation.

To characterize the biochemical activities of the 11 *est1*^−^ mutations identified by ODN mutagenesis, we examined how each of these mutations affected binding to two known Est1-binding partners, Est3 and Cdc13. We previously showed that Est1 contains a binding site for the Est3 telomerase subunit, located in the N-terminal domain of Est1 in a region distinct from the RBD (Figure 2C; Tucey and Lundblad 2014); this Est1-Est3 interaction was defined by the *est1*-R269E mutation, which was recovered in our ODN screen (Figure 1 and Figure S2 in File S1). Although the Est1-R269E mutant protein formed a preassembly complex that was indistinguishable from wild-type (Figure 2B), subsequent association of Est3 with the mutant Est1-R269E-TLC1-Est2 subcomplex to form the telomerase holoenzyme was reduced (Tucey and Lundblad 2014; Figure S5 in File S1). To ask if additional *sof*^−^ mutations identified by ODN mutagenesis affected the interaction between Est1 and Est3, we integrated *sof*^−^ alleles into an Est1-(myc)_12_ Est3-(FLAG)_3_ strain and examined the interaction between Est1 and Est3 following anti-FLAG IPs. This identified a cluster of three mutations in the C-terminal region of Est1 that were substantially impaired for the Est1-Est3 interaction (Figure 4A). The severity of this biochemical defect was reflected in the *in vivo* phenotype of the mutant strains bearing these three alleles, which exhibited critically short telomeres in all three cases (Figure 1E). Notably, other *sof*^−^ mutations in the C-terminal domain with pronounced *in vivo* phenotypes had no impact on the Est1-Est3 association (Figure 4B). This Est1-Est3 association was also dependent on prior formation of the Est1-TLC1-Est2 subcomplex; if Est1 was unable to bind the TLC1 RNA due to a mutation in the Est1 RBD, the Est1-Est3 interaction was abolished (Figure 4C). These results, combined with the identification of the RBD in Figure 3, show that the Est1 protein contains three distinct binding sites that are required for the formation of the quaternary enzyme complex: the RBD in the extreme N-terminus of Est1, and a bimodal Est3-binding interface that employs Est3-interacting residues in both the N- and C-terminal halves of Est1.

### An expanded Est1 interface is required for the interaction with Cdc13

**Figure 5:**
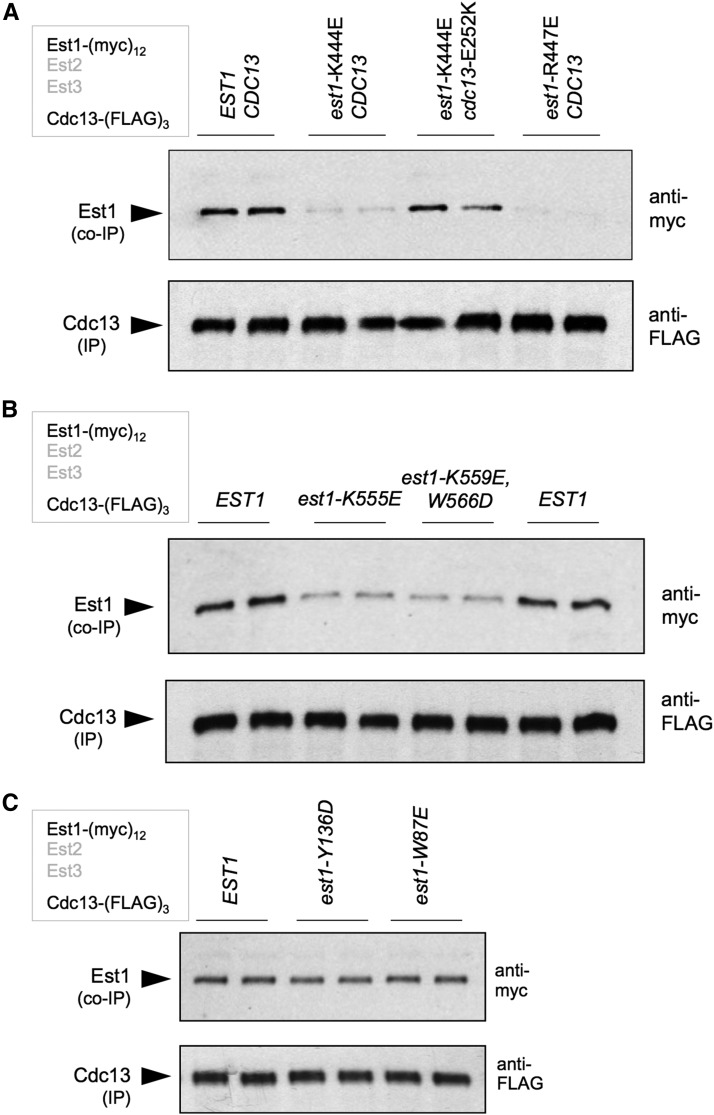
An expanded interface on Est1 is required for binding to Cdc13. (A) Association between Est1 and Cdc13, monitored by anti-FLAG IP, with Est1 and Cdc13 tagged with (myc)_12_ and (FLAG)_3_ epitopes, respectively, was abolished by mutations in adjacent residues (*est1*-K444E and *est1*-R447E). Consistent with prior observations (Tucey and Lundblad 2013), the *est1*-K444E-dependent loss of interaction with Cdc13 was restored when combined with the *cdc13*-E252K mutation (aka *cdc13*-2). (B) The Est1-Cdc13 association, assessed as in (A), was impaired by additional mutations in an ∼10-amino acid span from 555 to 566 (since the *in vivo* defect displayed by the *est1*-K559E and *est1*-W566D single mutations was very modest, as shown in Figure 1F, these two mutations were combined). (C) The RNA-binding-defective Est1-W87E and Est1-Y136D proteins exhibited wild-type levels of association with Cdc13, as monitored by anti-FLAG IPs using the same protocol as in (A). co-IP, coimmunoprecipitation; IP, immunoprecipitation.

Mutations recovered from the ODN mutagenesis were also tested for whether they affected the interaction between Est1 and Cdc13, using a strain with (myc)_12_ and (FLAG)_3_ tags on Est1 and Cdc13, respectively. We have previously reported that an interaction between Est1 and Cdc13 can be detected in anti-Cdc13 immunoprecipitates. This biochemical association recapitulates the genetic interaction between these two proteins (Pennock *et al.* 2001), as Est1-Cdc13 binding is eliminated by the recruitment-defective *cdc13*-2 mutation, but restored in a *cdc13*-2 strain containing a cosuppressing *est1*-60 mutation (Tucey and Lundblad 2013; Figure 5A), providing strong support for a direct interaction between Est1 and Cdc13. The *est1*-60 mutation (*est1*-K444E) was also identified in our ODN-directed mutant screen, as well as a second mutation in an immediately adjacent residue (*est1*-R447E; Figure 1 and Figure S2 in File S1); both mutations strongly reduced the Est1-Cdc13 interaction (Figure 5A). An additional cluster of mutations (at amino acids 555–566) made a more modest contribution to the association between Est1 and Cdc13 (Figure 5B). As was observed for mutations that affected Est1-TLC1 and Est1-Est3 interactions, the extent of the Est1-Cdc13-binding defect in the *in vitro* assay (Figure 5) correlated well with the impact on telomere length *in vivo* (Figure 1).

In contrast to the Est1-Est3 interaction (Figure 4C), the association between Est1 and Cdc13 was not dependent on the Est1-TLC1 interaction, as two mutant Est1 proteins (Est1-W87E and Est1-Y136D) that were defective for RNA binding, and thus incapable of associating with the telomerase complex (Figure 3C), still bound Cdc13 at wild-type levels (Figure 5C). Reciprocally, the Est1-Est3 and Est1-Cdc13 interactions were not interdependent, as mutant Est1 proteins that could not bind Cdc13 (Est1-K444E and Est1-R447E) were able to form the Est1-TLC1-Est2 preassembly complex (Figure 2B) and associate with Est3 to form the subsequent Est3-Est1-TLC1-Est2 holoenzyme (Figure 4B). Similarly, the mutant Est1-D510R and Est1-F511D proteins, which had lost association with Est3 (Figure 4A), were still capable of binding Cdc13 (Figure S5 in File S1), which is also consistent with a prior report showing that an Est1 mutant protein bearing a different ODN missense mutation in one of these residues (*est1*-F511S; Virta-Pearlman *et al.* 1996) still retains association with telomeric chromatin (Sealey *et al.* 2011). This provides strong support for a model in which assembly of the telomerase holoenzyme and the interaction between Cdc13 and Est1 are biochemically independent events, as evidenced by the fact that an interaction between Cdc13 and Est1 occurs in G1 phase (Tucey and Lundblad 2013), at a point when Cdc13 cannot be detected at telomeres and the holoenyzme has not yet assembled.

### A newly discovered fourth function for Est1

**Figure 6:**
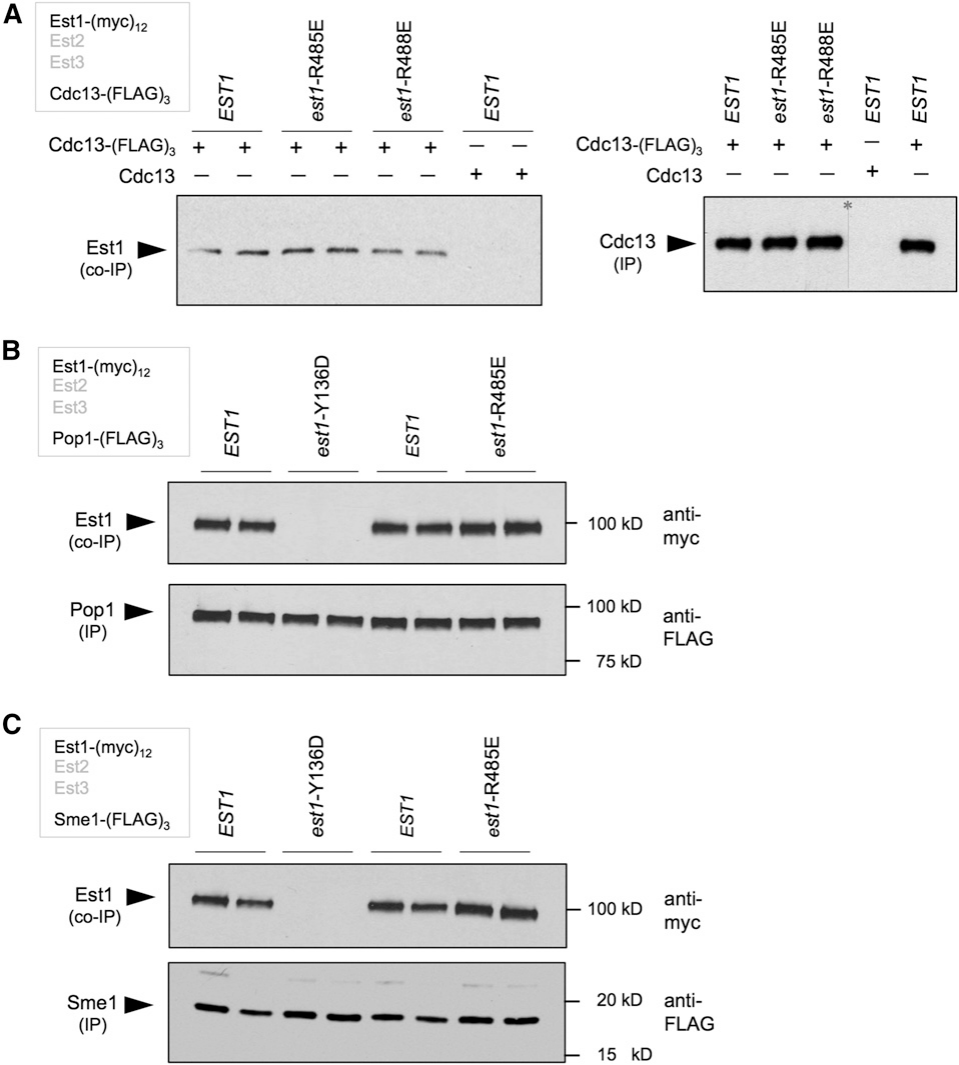
Two mutations in the C-terminal domain of Est1 define a fourth novel function. (A) The association between Est1 and Cdc13, monitored by anti-FLAG IP as in Figure 5A, is unaffected by the *est1*-R485E and *est1*-R488E mutations; an independent repeat of this experiment for *est1*-R488E is shown in Figure S5 in File S1. (B and C) Association between Est1 and Pop1 or Sme1 was monitored by anti-FLAG IP, with Est1 tagged with (myc)_12_ and Pop1 or Sme1 tagged with (FLAG)_3_ epitopes. Co-IP of Est1 with either the Pop complex (B) or the Sm complex (C) was abolished by the TLC1-binding-defective *est1*-Y136D mutation, but unaffected by the *est1*-R485E mutation. co-IP, coimmunoprecipitation; IP, immunoprecipitation.

The analysis of the effects of these 11 *sof*^−^ mutations on binding to TLC1, Est1, or Cdc13 identified mutations in two closely spaced amino acids (*est1*-R485E and *est1*-R488E) that were unaffected for interaction with these three well-characterized binding partners of Est1. The mutant Est1-R485E and Est1-R488E proteins were indistinguishable from the wild-type Est1 protein in their ability to associate to form the Est1-TLC1-Est2 preassembly complex (Figure 2B), to bind Est3 to form the holoenzyme (Figure 4B), or to interact with the Cdc13 recruitment factor (Figure 6A). Furthermore, the telomerase disassembly pathway was unperturbed in strains bearing either of these two mutations (Figure S6 in File S1 and data not shown). The robust ODN phenotype exhibited by these two mutations was also reversed by the presence of an RNA-binding-defective allele (Figure S6 in File S1), arguing that Est1 performs this fourth activity as a component of the telomerase holoenzyme. These data, combined with the strong *in vivo* phenotype displayed by the *est1*-R485E and *est1*-R488E strains (Figure 1D), argues that telomerase has a fourth Est1-dependent activity that is critical for telomere replication.

Numerous factors besides TLC1, Cdc13, or Est3 have been proposed as direct interactors with either Est1 or the telomerase holoenzyme, and thus could be candidates that mediate this fourth activity. The strength of the *in vivo* phenotype displayed by the *est1*-K485E and *est1*-K488E strains suggests that a defect in this proposed factor will also exhibit an Est^−^ phenotype (*i.e.*, critically short telomeres and an accompanying senescence phenotype). This prediction potentially rules out candidates encoded by nonessential genes, since the only nonessential genes that have an Est phenotype when mutated are previously identified telomerase components (encoded by *EST1*, *EST2*, *EST3*, and *TLC1*; Askree *et al.* 2004). Therefore, we turned our attention to two previously described telomerase-interacting complexes encoded by essential genes: the Sm complex (Seto *et al.* 1999) and the Pop protein complex (Lemieux *et al.* 2016). Strains bearing a (myc)_12_ tag on Est1 and a (FLAG)_3_ tag on a subunit of either of these two complexes (Pop1 or Sme1) were constructed and subjected to anti-FLAG IP. As shown in Figure 6, B and C, both the Pop1 and Sme1 proteins exhibited a robust co-IP association with Est1. However, this co-IP signal was abolished if Est1 was unable to bind the TLC1 RNA, due to the introduction of the TLC1-binding-defective *est1*-Y136D mutation (Figure 6, B and C). Furthermore, the interaction between telomerase and the Sm and Pop complexes was not affected in *est1*-R485E and *est1*-R488E strains (Figure 6, B and C and data not shown). Thus, the association of the Sm and Pop complexes with telomerase is not mediated through a direct interaction with Est1.

We also reexamined proteins encoded by nonessential genes, which have been proposed to physically associate with yeast telomerase, using co-IP (Figure S6 in File S1). Somewhat surprisingly, we failed to detect an interaction between telomerase and a number of factors previously proposed to associate with telomerase (Pif1, Ebs1, and Sir4), including proteins that have been proposed to directly interact with Est1 (Yku80 and Msp3).

### Reexamining whether Est1 is regulated by proteasome-mediated degradation

Several prior studies have shown that Est1 protein levels are regulated through the cell cycle (Osterhage *et al.* 2006; Tucey and Lundblad 2013). In G1 phase, Est1 and Est2 protein levels are equivalent, but as cells progress into S phase, Est1 protein levels increase by almost threefold, whereas Est2 protein levels remain constant; nevertheless, the Est1-TLC1-Est2 subcomplex, which forms early in S phase with Est1 and Est2 in a 1:1 ratio, remains unchanged through the rest of the cell cycle (Tucey and Lundblad 2013). This cell cycle-dependent regulation of Est1 protein levels has been proposed to rely on proteasome-mediated degradation, whereby targeted ubiquitination of Est1 contributes to telomere homeostasis (Osterhage *et al.* 2006; Ferguson *et al.* 2013; Lin *et al.* 2015). This predicts that one or more lysine residues in Est1 should be substrates for ubiquitination, with consequences for telomere length regulation if ubiquitination is blocked. However, despite the inclusion of a large number of lysine residues in our ODN screening strategy (Figure S1 in File S1), only three lysines (K444, K555, and K559) were recovered that, when mutated, exhibited an effect on telomere length maintenance. Based on our biochemical analysis, these three lysine residues mediate the interaction between Est1 and Cdc13 (Figure 5); furthermore, since Est1 protein levels and assembly of the Est1-TLC1-Est2 subcomplex were unaffected in these three mutant strains (Figure 2), this argues that none of these three lysines were involved in proteasome-mediated degradation of Est1.

**Figure 7:**
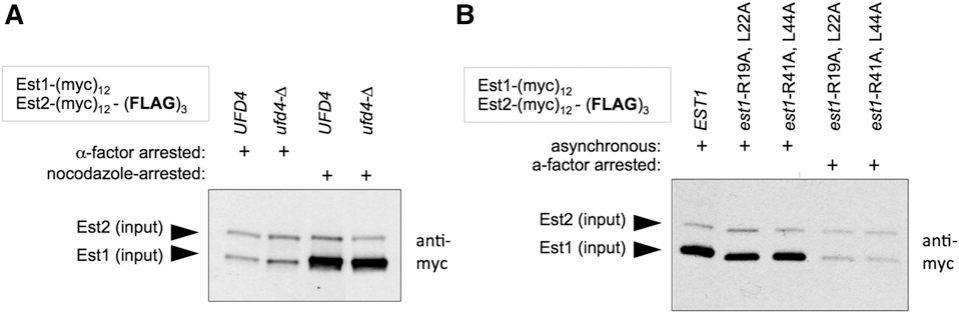
Reinvestigating the role of proteasomal degradation on Est1 protein levels. (A) The steady-state levels of Est1 and Est2, from extracts of cells arrested either in G1 or G2/M and assayed by anti-myc westerns, are unaffected by the loss of *UFD4*, which has been proposed to target Est1 for degradation (Lin *et al.* 2015). (B) A similar analysis of Est1 and Est2 protein levels, assayed as in (A), does not reveal any changes in response to mutations in previously proposed Destruction boxes (Ferguson *et al.* 2013).

However, lysines that are targets for ubiquitination might be overlooked by an ODN strategy. To address this possibility, we examined the effect on telomere length of mutations in every lysine residue that was conserved in ≥3 species within the *Saccharomyces sensu stricto* group (a closely related set of species descended from a common ancestor that underwent a whole-genome duplication; Wolfe and Shields 1997), using an LOF assay. As shown in Figure S3 in File S1, none of these mutant strains bearing K → A mutations exhibited any marked defects in telomere length. Although this single-residue mutational analysis does not rule out the possibility that modification at multiple lysines is required, we were also unable to detect a change in Est1 protein levels in response to a defect in Ufd4 (Figure 7A), which was suggested to be the E3 ubiquitin ligase that targets Est1 for degradation (Lin *et al.* 2015). Several candidate Destruction boxes in Est1 have also been proposed to mediate the interaction between the anaphase-promoting complex (APC) and Est1 (Ferguson *et al.* 2013). However, when steady levels of the Est1 protein, normalized to Est2, were monitored in strains in which these candidate APC recognition sites in Est1 were eliminated, we did not observe any change in Est1 protein levels in extracts prepared from either cells arrested in G1 or from asynchronous cultures (Figure 7B). Collectively, the above results challenge the premise that Est1 is a target of ubiquitination-dependent proteasomal degradation during the G1 phase of the cell cycle. We suggest instead that the almost threefold increase in Est1 protein during the cell cycle is the result of the previously observed almost threefold increase in Est1 mRNA levels between G1 and G2/M (Spellman *et al.* 1998; Osterhage *et al.* 2006).

## Discussion

**Figure 8:**
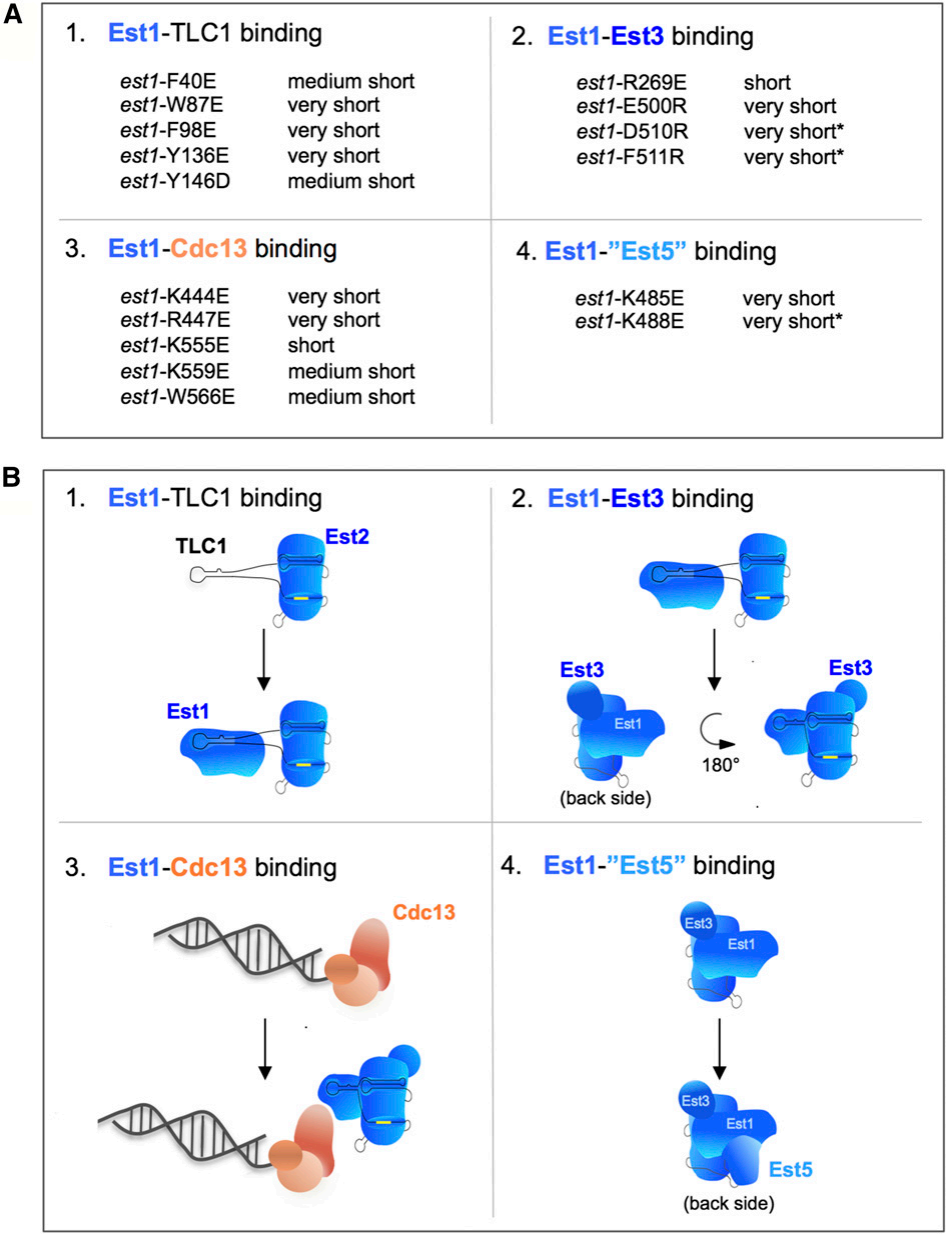
A working model for Est1. (A) A summary of the *sof*^−^ mutations identified by either loss-of-function approaches (category 1) or overexpression dominant negative approaches (categories 2–4) and the effect of these mutations on telomere length and senescence (indicated by an asterisk). (B) A schematic depiction of the biochemical activity that is disrupted by each of the four categories of *sof*^−^ mutations analyzed in this study. In panel 1, a simplified version of TLC1 is used to illustrate that Est1 and Est2 have independent binding sites on the telomerase RNA (Livengood *et al.* 2002); the template region of the RNA is indicated by a yellow box. The artistic rendition in panel 2 shows the association of Est3 with domains on both Est1 and Est2 to form the holoenzyme, accompanied by a proposed conformational change in the telomerase complex that is induced upon Est3 binding. Panels 3 and 4 show association of proteins encoded by known (*CDC13*/*EST4*) and proposed (*EST5*) essential genes; we currently have no information to assess whether the proposed fourth function in panel 4 occurs before (as shown) or after the telomerase recruitment depicted in panel 3.

In this study, we have generated a large panel of *sof*^−^ mutations in *EST1*, which we show to define four biochemically distinct activities. Two of these activities mediate telomerase assembly; formation of the Est1-TLC1-Est2 preassembly complex relies on a novel RBD in the N-terminus of the Est1 protein, and subsequent formation of the telomerase quaternary complex requires Est3 interaction sites located in both the N- and C-terminal halves of the Est1 protein. A third activity promotes telomerase recruitment, through a direct interaction between Est1 and Cdc13. Finally, we describe a fourth newly discovered role for Est1, based on a cluster of mutations that do not affect either telomerase assembly or recruitment. Since at least one *sof*^−^ mutation associated with each of these four activities confers critically short telomeres, this indicates that each of these four activities is indispensable for Est1’s contributions to telomere length maintenance. The collection of *sof*^−^ mutations analyzed in this study, along with their assigned biochemical activities, are summarized in Figure 8A.

### Are there more than four Est1 activities?

An obvious question raised by this analysis is whether our genetic strategy was in fact comprehensive, particularly since our mutagenesis was biased toward several amino acid categories. This question is addressed, at least in part, by a comparison with a prior ODN screen of another telomerase subunit (Est3) that relied on the same mutation bias. The resulting collection of *sof*^−^ alleles of *EST3* defined two biochemically distinct activities (Lee *et al.* 2008; Lubin *et al.* 2013) that were subsequently shown to map to two clusters of residues on the Est3 protein surface, dubbed the TEL and TELR patches (Rao *et al.* 2014). Once the Est3 structure became available, a structure-guided mutagenesis of the complete Est3 protein surface uncovered only two additional residues that conferred a strong telomere replication defect when mutated (in both LOF and ODN assays), which also mapped to the TEL and TELR patches (Rao *et al.* 2014). Thus, even with a bias toward charged residues, the ODN screen of *EST3* successfully identified the two functions performed by this telomerase subunit.

We also subjected *EST1* to a very high level of mutagenesis: 165 *est1*^−^ missense mutations were screened for either ODN or LOF phenotypes, with 60% screened in both phenotypic assays; this represents an average coverage of one mutation for every four to five residues. Nevertheless, several regions of Est1 (amino acids 273–400 and 570–699) were dispensable for telomere length maintenance (Figure 2C), which potentially reveals omissions in our genetic strategy. Alternatively, this may reflect the fact that. in *Saccharomyces cerevisiae*, there is a second gene, called *EBS1*, which is highly similar to *EST1*; these two paralogs arose as the result of the whole-genome duplication prior to the evolution of the *Saccharomyces* clade (Dujon 2010). In these species, Est1 and Ebs1 perform nonoverlapping roles, as a subunit of telomerase (Tucey and Lundblad 2013) or as a component of the nonsense-mediated decay (NMD) pathway (Ford *et al.* 2006; Luke *et al.* 2007), respectively. In contrast, in most species (such as fission yeast), there is only a single protein that is homologous to the *EST1* and *EBS1* paralogs; in *Kluyveromyces lactis*, a deletion of this gene confers defects in both telomerase function and NMD (Hsu *et al.* 2012). This argues that, in *S. cerevisiae*, the Est1 and Ebs1 proteins presumably contain unique features that dictate their nonoverlapping *in vivo* roles. If so, this may account for why several regions of Est1 appear to be unnecessary for function (Figure 2C). We are testing this premise by conducting a comparable comprehensive mutagenesis of the *S. cerevisiae EBS1* gene.

Our collection of *sof*^−^ alleles does not include mutations in a number of residues that have been reported in prior publications (Evans and Lundblad 2002; Zhang *et al.* 2010; Sealey *et al.* 2011; Tong *et al.* 2011; Hawkins and Friedman 2014), either because we could not reproduce the original mutant phenotype (Figure S3 in File S1) or because our analysis argued that the mutant phenotype was due, at least in part, to protein destabilization (Figure S1 in File S1). This latter point might be attributed to the fact that several of these prior studies analyzed clusters of mutations (including mutant isolates from our laboratory; Evans and Lundblad 2002), which increases the possibility that one or more amino acids in a cluster might not be solvent-accessible.

### A working model for Est1

We propose a simple framework for the four Est1 activities (Figure 8B). Early in the cell cycle, Est1 employs its RBD to form an Est1-TLC1-Est2 subcomplex (Figure 3 and panel 1 in Figure 8B; Tucey and Lundblad 2013). This subcomplex subsequently binds the Est3 telomerase subunit to form the telomerase quaternary complex late in the cell cycle (Tucey and Lundblad 2014; panel 2 in Figure 8B). Est3 loading is a surprisingly complex step in the telomerase assembly pathway, as it involves two different sites on the N- and C-terminal domains of Est1 as well as the Ten domain of Est2 (Figure 4; Tucey and Lundblad 2014). We have previously suggested that association of Est3 might confer a conformational change in the telomerase complex; if so, this might explain why multiple surfaces on the preassembly complex are involved in Est3 binding. Once the holoenzyme telomerase complex has been assembled, we propose that Est1 carries out two distinct functions as a subunit of telomerase (panels 3 and 4, Figure 8B). The first of these is the well-studied role in telomerase recruitment, whereby Est1 provides a bridge between the telomere-bound t-RPA complex and the catalytic core of telomerase, through a presumably direct interaction with Cdc13 (Pennock *et al.* 2001; Bianchi *et al.* 2004; Tucey and Lundblad 2013). Est1 also performs a newly discovered activity that is critical for telomere length maintenance, which does not appear to be mediated by previously characterized candidate telomerase interactors (Figure 6, B and C and Figure S6 in File S1). We speculate that the function of Est1 that is disrupted by mutations in Arg485 and Arg488E might be an interaction with an as-yet-undiscovered protein encoded by an essential gene (hypothetically dubbed “Est5” in Figure 8B), analogous to the interaction between Est1 and the product of the essential *CDC13* gene (which was originally called *EST4*; Lendvay *et al.* 1996). The discovery of this fourth function also illustrates the efficiency and effectiveness of the ODN screening methodology, which was capable of discovering a new regulatory role even for a well-studied protein like Est1.

This analysis also highlights the potential limitations of using complete gene deletions for functional analysis, as an *est1*-∆ deletion simultaneously blocks assembly of the Est3 subunit into the complex, telomerase recruitment, and a newly identified regulatory function. Nevertheless, impairment of these distinct Est1 activities all result in a common phenotype: critically short telomeres (Figure 1, D–G and summarized in Figure 8A). This phenotypic similarity masks the pleiotropic consequences of many types of experiments, such as epistasis analysis, which monitors the consequences of combining an *est1*-∆ null mutation with mutations in other genes. In future analyses, we suggest that the employment of a representative set of *sof*^−^ mutations has the potential to uncover genetic interactions that are specific for individual functions of a nonessential gene, thereby providing a far more nuanced view of the complex network of interactions that occur inside the cell.

## 

## Supplementary Material

Supplemental material is available online at www.genetics.org/lookup/suppl/doi:10.1534/genetics.117.300145/-/DC1.
