# Supplementary material for: Using Separation-of-Function Mutagenesis To Define the Full Spectrum of Activities Performed by the Est1 Telomerase Subunit in Vivo
Source: Genetics. 2017 Nov 29;208(1):97–110. doi: 10.1534/genetics.117.300145 (PMC5753878; doi:10.1534/genetics.117.300145)
Supplement: Supplementary file 1 [file 97FileS1.pdf]

Using separation-of-function mutagenesis to define the full spectrum of activities performed by the Est1 telomerase subunit *in vivo*

Johnathan W. Lubin, Timothy M. Tucey and Victoria Lundblad

Supplemental Figures and Tables

Figure S1. Mutations in *EST1* that were examined for ODN and/or LOF phenotypes.

Figure S2. ODN phenotypes of 11 separation-of-function mutations in *EST1*

Figure S3. Loss-of-function analysis of telomere length for an extensive panel of *est1*¯ mutations

Figure S4. Additional analysis of the RNA binding domain of Est1

Figure S5. Additional analysis of Est3-binding-defective alleles of *EST1*

Figure S6. Additional analysis of the fourth function of Est1

Table S1. Yeast strain list

Table S2. Plasmid list

Supplemental Fig. S1
Lubin et al.

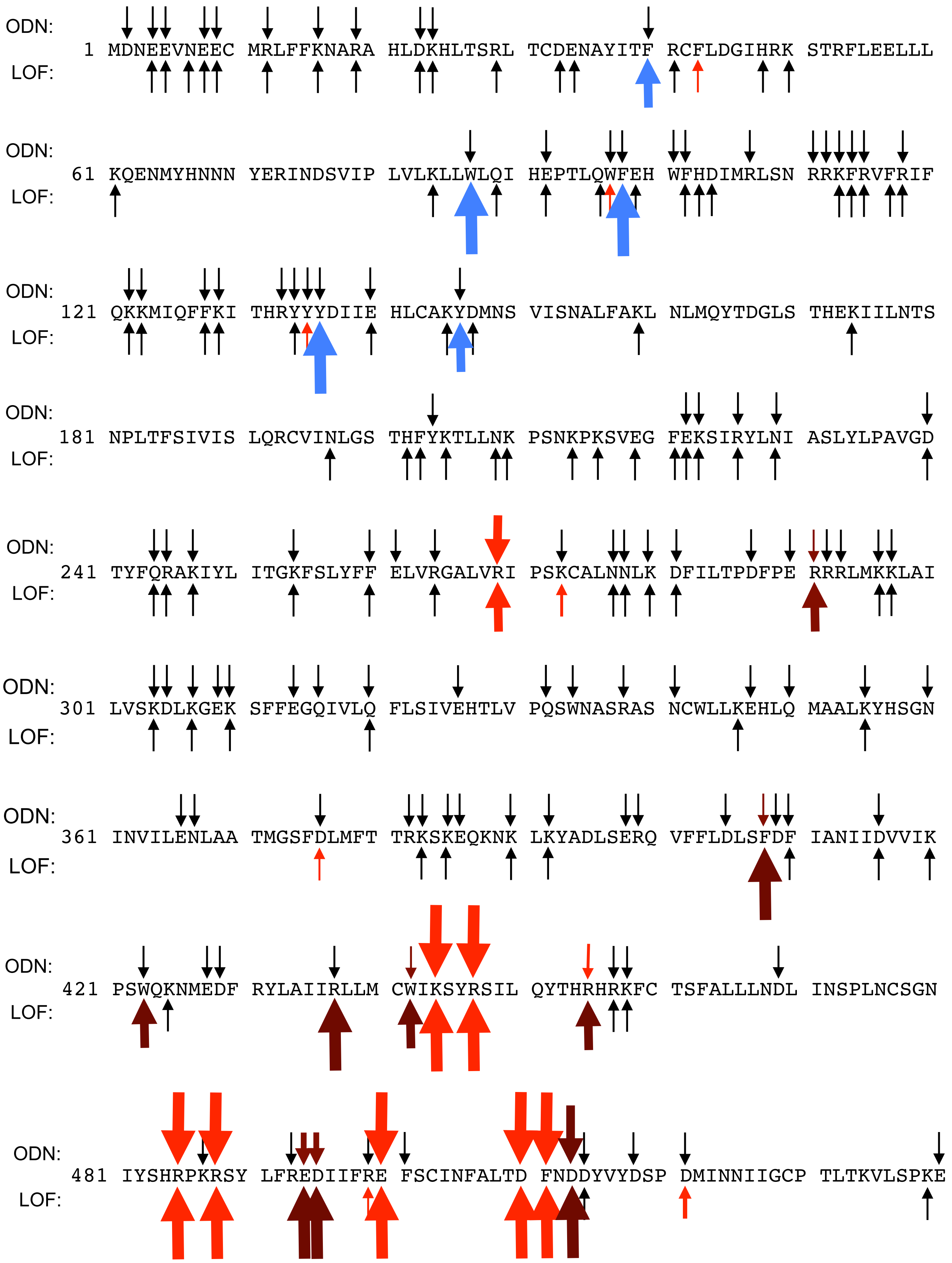

Supplemental Fig. S1 (continued)
Lubin et al.

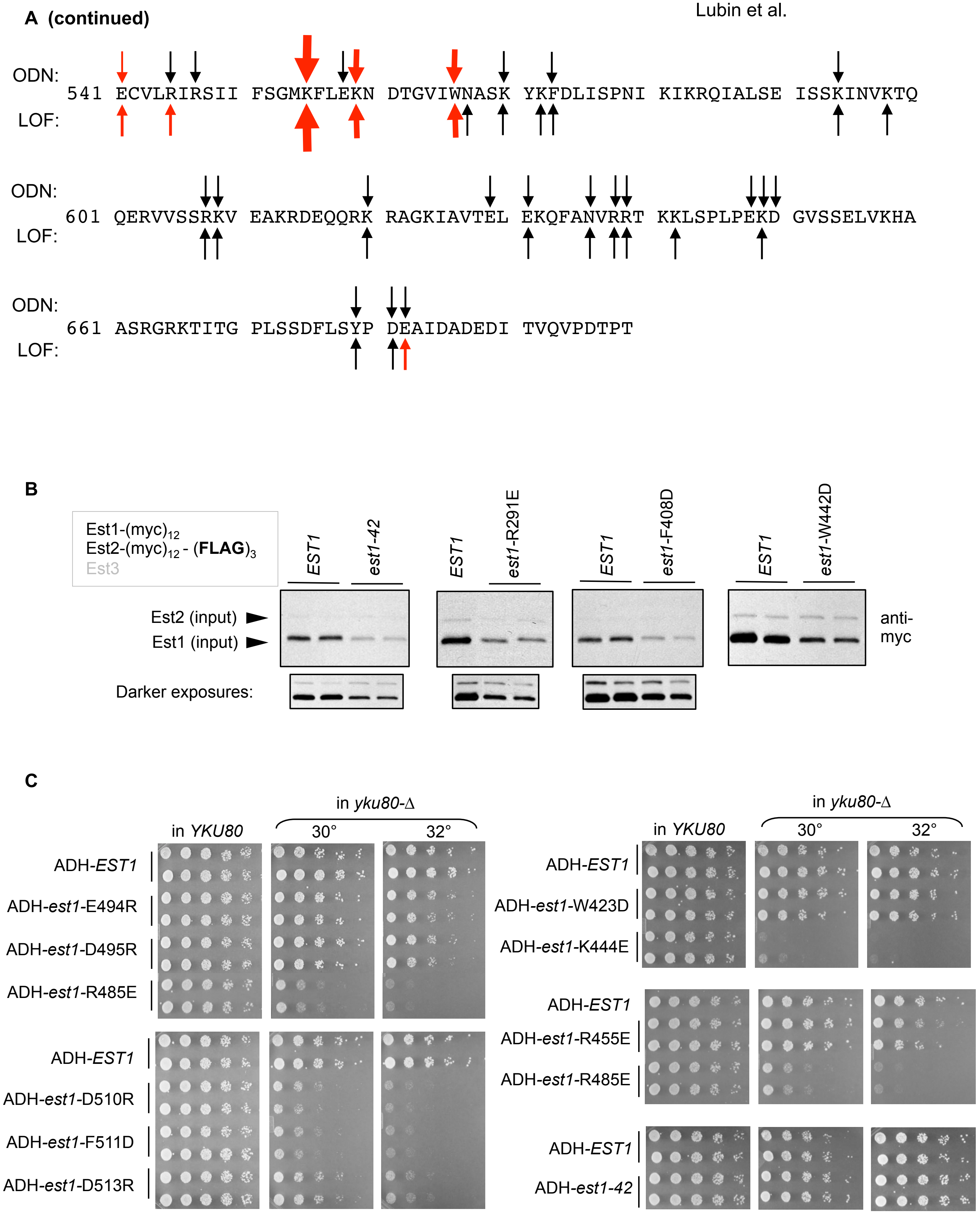

Supplemental Fig. S1 (continued)
Lubin et al.

D

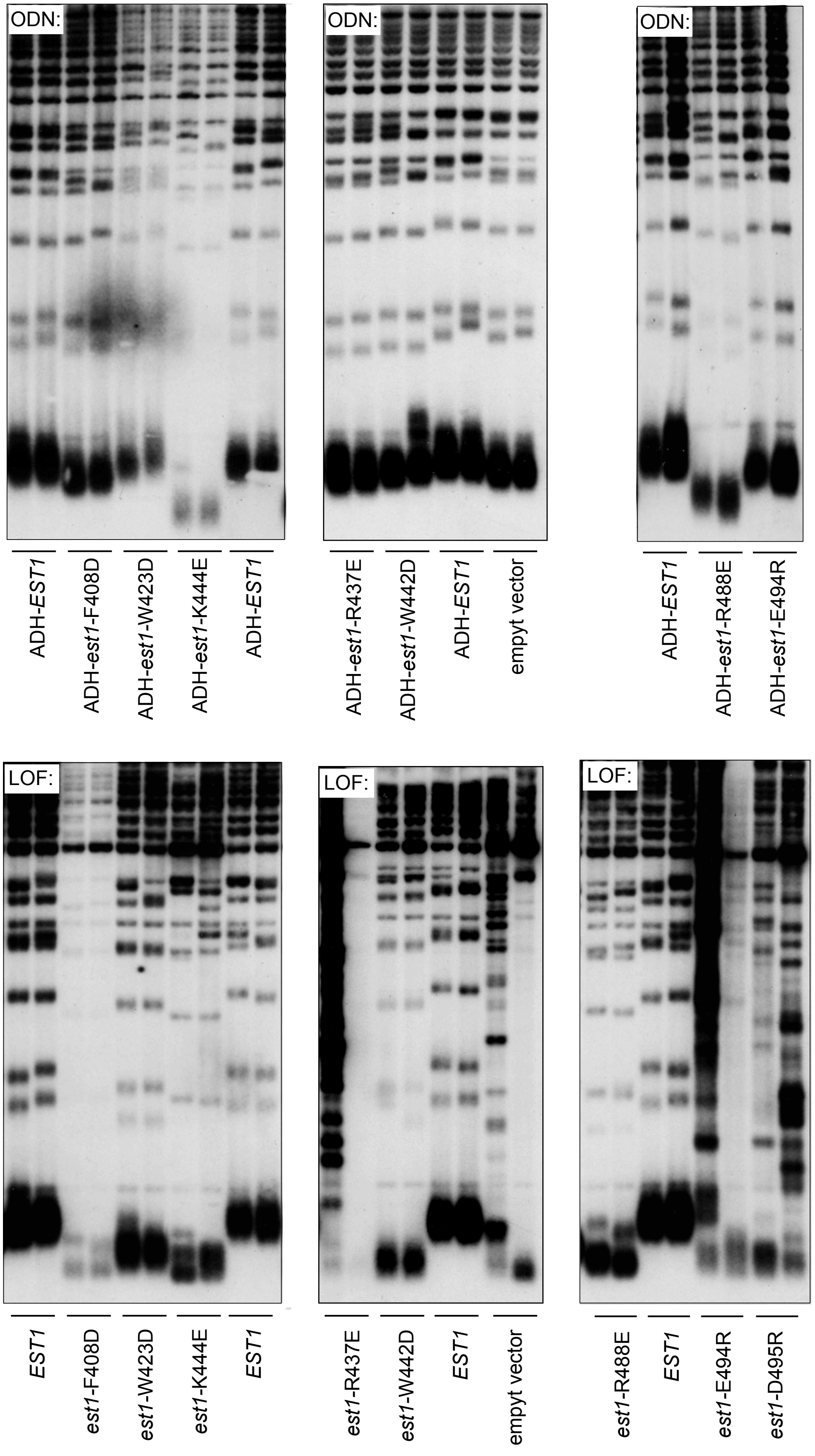

Figure S1. Mutations in *EST1* that were examined for ODN and/or LOF phenotypes.

(**A**) Schematic representation of ODN and/or LOF phenotypes resulting from mutations in individual amino acids, as indicated on the Est1 protein sequence. Examples of ODN phenotypes are shown in Figure 1A – 1C and Supplemental Figure S1C, S1D and S2, and examples of LOF phenotypes are shown in Figure 1D – 1G and Supplemental Figure S1D and S3. Mutations that failed to show a phenotype are indicated by a black arrow; mutations that conferred a phenotype are indicated by arrows in a range of sizes to indicate the severity of the phenotype. Brown arrows highlight those allele where the LOF phenotype was more severe than the ODN phenotype (see also Supplemental Figure S1B and S1C); blue arrows correspond to the five mutations that are impaired for binding to TLC1.

(**B**) Assessment of Est1 protein levels, for mutant Est1 proteins that exhibited LOF phenotypes that were more severe than the corresponding ODN phenotypes (ODN and LOF data for these alleles are shown in Supplemental Figure S1C, S1D and S3K). Mutant Est1 protein levels were reduced to ~25 to 30%, relative to wild type Est1, for the Est1-R291E and Est1-F408D proteins, and 2-fold for Est1-W442D; protein levels were assayed in strains in which each mutation was integrated into the genome, to avoid plasmid-dependent variations in Est1 protein levels, and the anti-myc signal for Est1-myc_{12}, was normalized to myc_{12}-Est2. The Est1-42 mutant protein, with amino acid changes (D287A, E290A, R291A, R292A) that partially overlapped with Est1-R291E, also showed a 4-fold reduction in protein levels. Genetic analysis of the *est1*-42 mutation was previously used to argue that this mutation was specifically impaired for the so-called "second function" of Est1 (Evans and Lundblad 2002); however, the results shown here argue that this "second function" phenotype was instead most likely due to reduced protein stability, rather than loss of a specific biochemical activity. A similar caveat applies to the interpretation of the in vivo role of Arg291 from a different study (Hawkins and Friedman 2014).

Evans, S. K. and V. Lundblad, 2002 The Est1 subunit of *Saccharomyces cerevisiae* telomerase makes multiple contributions to telomere length maintenance. Genetics 162: 1101-1115.

Hawkins, C. and K. L. Friedman, 2014. Normal telomere length maintenance in *Saccharomyces cerevisiae* requires nuclear import of the ever shorter telomeres 1 (Est1) protein via the importin alpha pathway. Eukaryot. Cell 13: 1036-1050.

Figure S1 (continued). Mutations in *EST1* that were examined for ODN and/or LOF phenotypes.

(**C**) Examples of mutations that exhibit weak ODN phenotypes in the *yku80*-Δ synthetic lethal assay (relative to the magnitude of the corresponding LOF phenotype). A *yku80*-Δ / p *CEN URA3 YKU80* strain was transformed with 2 μ plasmids containing the indicated *est1*¯ mutation, which was expressed by the constitutive ADH promoter; serial dilutions of two independent transformants for each mutation were examined for viability on media that selected for the presence or loss of the *YKU80* plasmid. The temperature-dependence of this synthetic growth phenotype provides a sensitive assay for detecting a broad range of ODN phenotypes, as previously described (Lee *et al*. 2008) and as illustrated by the *est1*-D513R mutation; this mutation confers an LOF phenotype that was indistinguishable from that conferred by *est1*-D510R and *est1*-F511D (Figure 1E and data not shown), but an ODN phenotype that was reproducibly less pronounced, relative to that of *est1*-D510R and *est1*-F511D.

Lee, J., Mandell, E. K., Tucey, T. M., Morris, D. K., and V. Lundblad, 2008. The Est3 protein associates with yeast telomerase through an OB-fold domain. Nat. Struct. Mol. Biol. 15: 990-997.

(**D**) Telomere length analysis in ODN and LOF assays, for six mutations that exhibit an attenuated ODN phenotype when compared with the corresponding LOF phenotype; *est1*-K444E and *est1*-R488E, which exhibit comparably strong ODN and LOF phenotypes, were included for comparison. ODN or LOF effects on telomere length (the top three panels and the bottom three panels, respectively) were assayed as described in Figure 1.

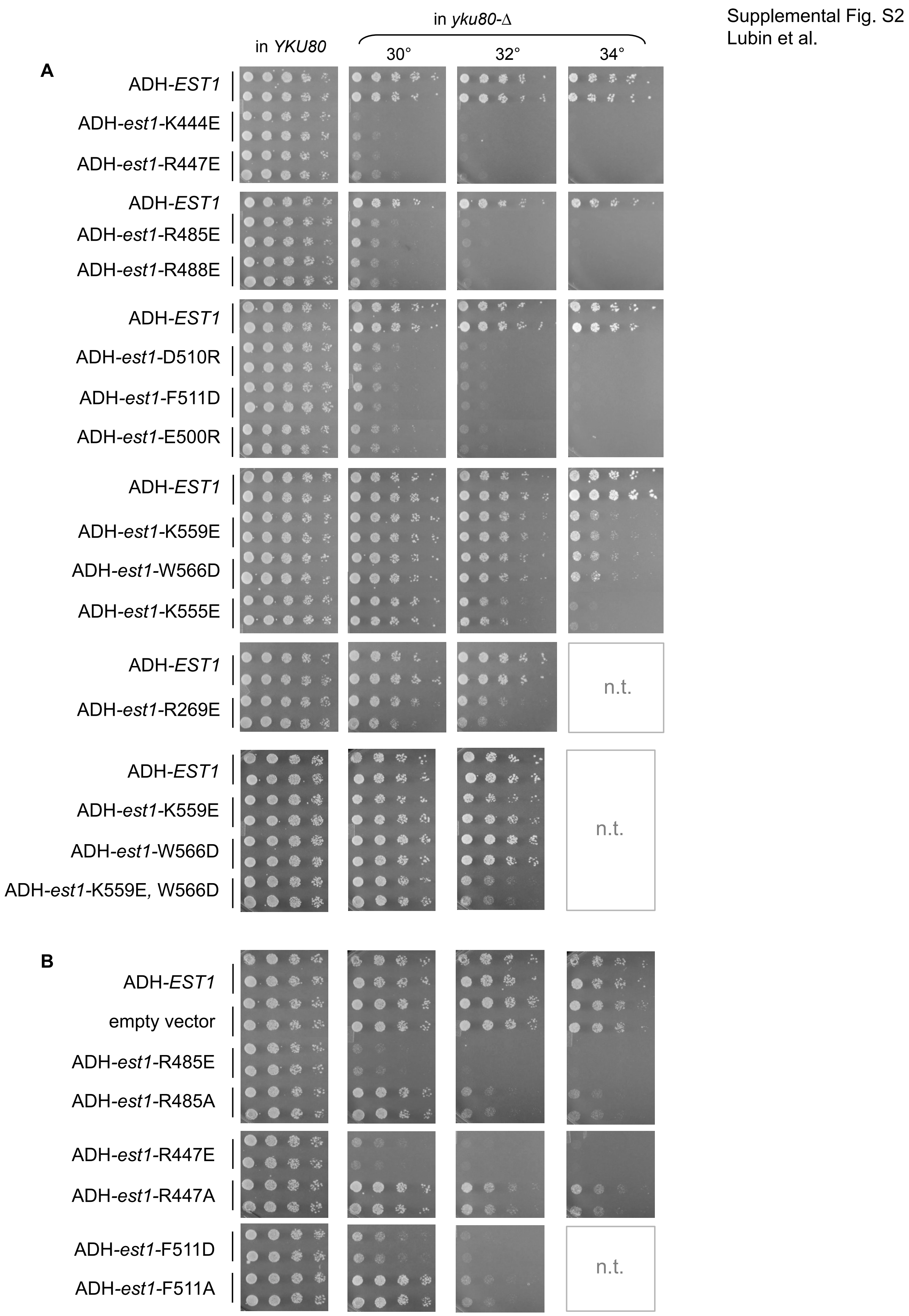

Supplemental Fig. S2
Lubin et al.
in YKU80
in yku80-Δ
30°
32°
34°
A
ADH-EST1
ADH-est1-K444E
ADH-est1-R447E
ADH-EST1
ADH-est1-R485E
ADH-est1-R488E
ADH-EST1
ADH-est1-D510R
ADH-est1-F511D
ADH-est1-E500R
ADH-EST1
ADH-est1-K559E
ADH-est1-W566D
ADH-est1-K555E
ADH-EST1
ADH-est1-R269E
n.t.
ADH-EST1
ADH-est1-K559E
ADH-est1-W566D
ADH-est1-K559E, W566D
n.t.
B
ADH-EST1
empty vector
ADH-est1-R485E
ADH-est1-R485A
ADH-est1-R447E
ADH-est1-R447A
ADH-est1-F511D
ADH-est1-F511A
n.t.

Figure S2. ODN phenotypes of 11 separation-of-function mutations in *EST1*

(**A**) ODN phenotypes in a *yku80*-Δ strain of the 11 separation-of-function *est1*$^{-}$ mutations shown in Figure 1, assayed as described in Supplemental Figure S1C.

(**B**) Examples of three Est1 amino acids (Arg447, Arg485 or Phe511) that exhibited a pronounced ODN phenotype when mutated to a charged residue, but a much less severe ODN phenotype when mutated to alanine, consistent with our prior observations (Lubin et al. 2013).

Supplemental Fig. S3
Lubin et al.

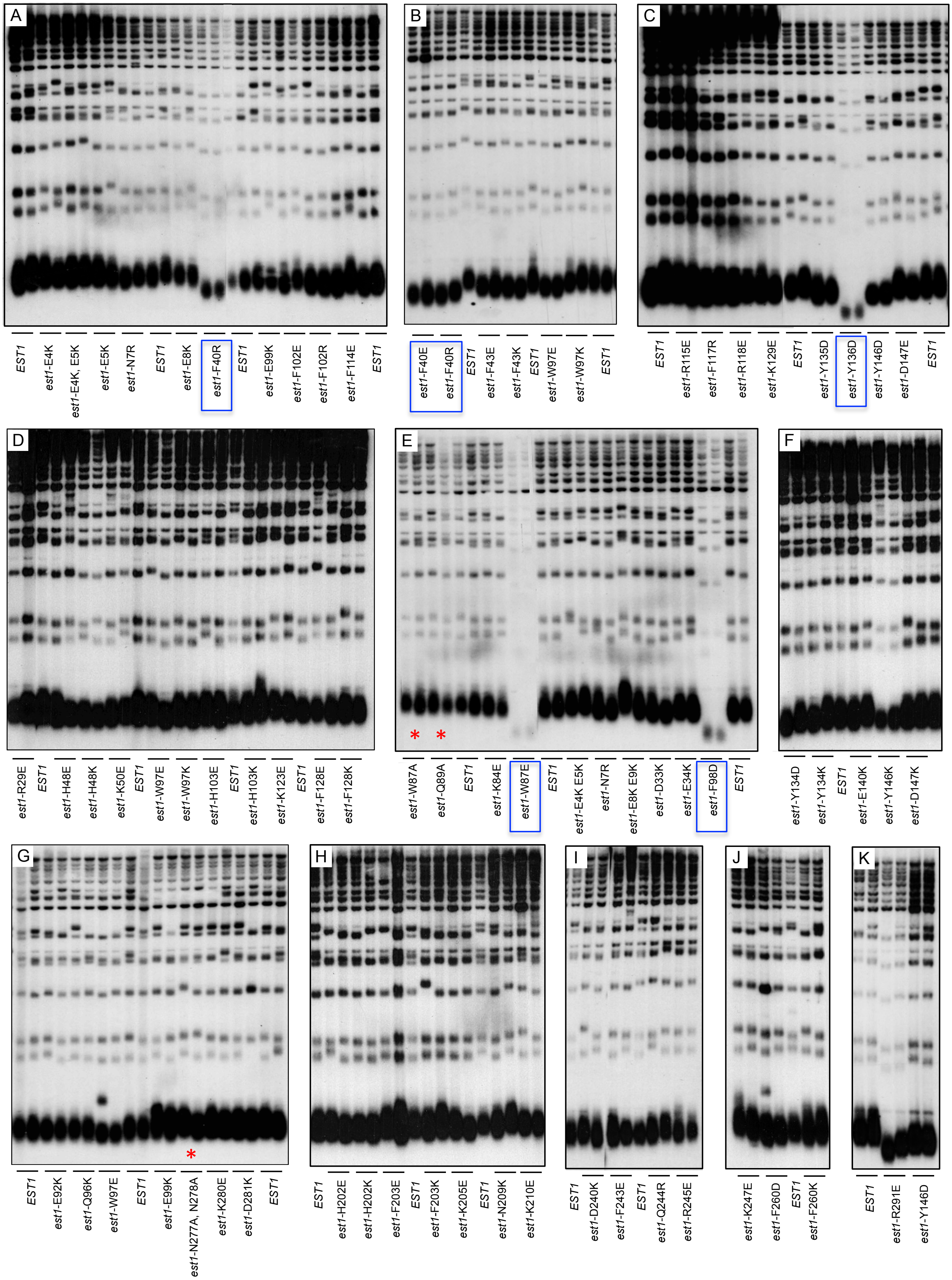

Supplemental Fig. S3 (continued)
Lubin et al.

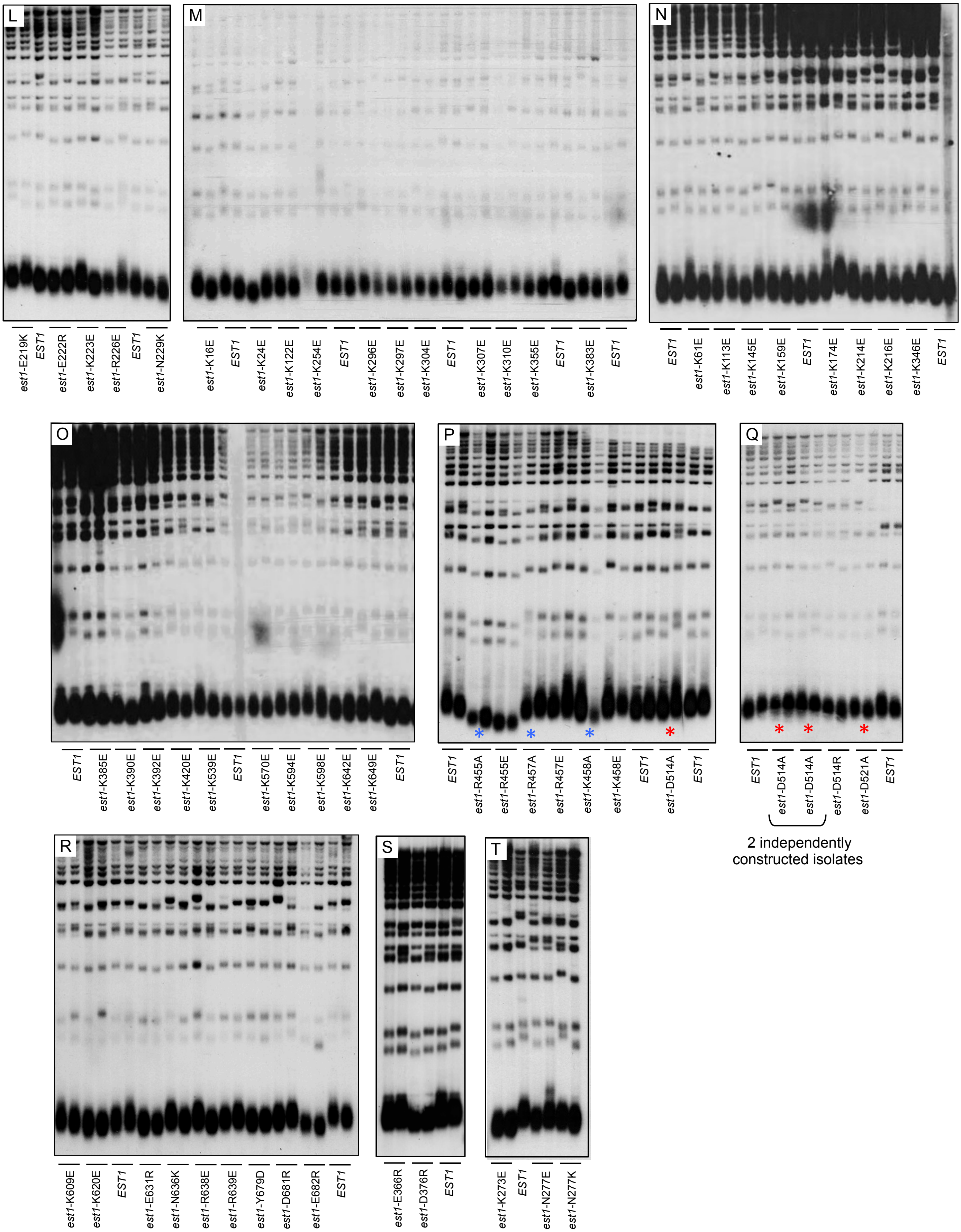

Figure S3. Loss-of-function analysis of telomere length for an extensive panel of *est1*¯ mutations.

Telomere length of *est1*-Δ strains transformed with single copy plasmids with the indicated *est1*¯ mutations, expressed by the *EST1* promoter, determined after ~75 generations of growth following transformation of the *est1*-Δ strain. Mutations that are boxed (panels A – E) are defective for RNA binding, as shown in Fig. 3. Mutations indicated by a red asterisk (panels E, G, P and Q) conferred a wild type telomere length phenotype, in contrast to what was observed in previous reports (Zhang *et al.* 2010; Tong *et al*. 2011; Sealey *et al.* 2011). Mutations with a blue asterisk (panel P) correspond to a cluster of residues proposed to mediate nuclear localization (Hawkins and Friedman 2014); we propose instead that the mutant phenotype more likely due to protein destabilization, based on the very weak ODN phenotype of *est1*-K455E (see Supplemental Figure S1C), relative to the more pronounced LOF phenotype for this mutation.

Hawkins, C. and K. L. Friedman, 2014. Normal telomere length maintenance in *Saccharomyces cerevisiae* requires nuclear import of the ever shorter telomeres 1 (Est1) protein via the importin alpha pathway. Eukaryot. Cell 13: 1036-1050.

Sealey, D. C., Kostic, A. D., LeBel, C., Pryde, F., and L. Harrington, 2011. The TPR-containing domain within Est1 homologs exhibits species-specific roles in telomerase interaction and telomere length homeostasis. BMC Mol. Biol. 12: 45.

Tong, X.-J., Li, Q. J., Duan, Y. M., Liu, N. N., Zhang, M. L., and J. Q. Zhou, 2011. Est1 protects telomeres and inhibits subtelomeric Y'-element recombination. Mol. Cell. Biol. 31: 1264-1274.

Zhang, M. L., Tong, X.-J., Fu, X. H., Zhou, B. O., Wang, J., Liao, X. H., Li, Q. J., Shen, N., Ding, J., and J. Q. Zhou, 2010. Yeast telomerase subunit Est1p has guanine quadruplex-promoting activity that is required for telomere elongation. Nature Struct. Mol. Biol. 17: 202-209.

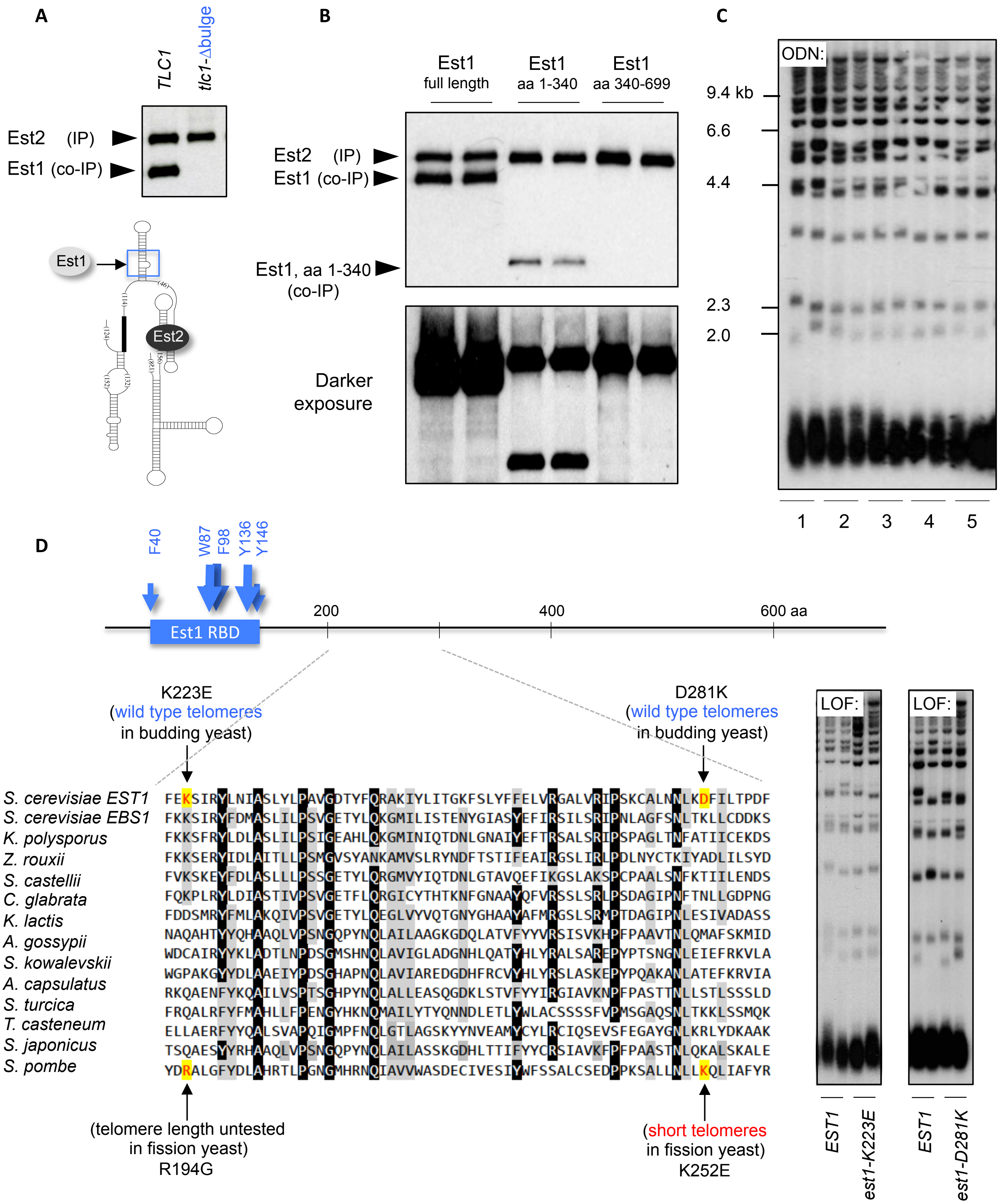
A
TLC1
tlc1-Δbulge
Est2 (IP)
Est1 (co-IP)
Est1
Est2
B
Est1 full length
Est1 aa 1-340
Est1 aa 340-699
Est2 (IP)
Est1 (co-IP)
Est1, aa 1-340 (co-IP)
Darker exposure
C
ODN:
9.4 kb
6.6
4.4
2.3
2.0
1
2
3
4
5
D
F40
W87
F98
Y136
Y146
Est1 RBD
200
400
600 aa
K223E
(wild type telomeres
in budding yeast)
D281K
(wild type telomeres
in budding yeast)
S. cerevisiae EST1 FEKSIRYLNIASLYLPAVGDTYFQRAKIYLITGKFSLYFFELVRGALVRIPSKCALNNLKDFILTPDF
S. cerevisiae EBS1 FKKSIRYFDMASLILPSVGETYLQKGMILISTENYGIASYEFIRSILSRIPNLAGFSNLTKLLCDDKS
K. polysporus FKKSFRYLDLASLILPSIGEAHLQKGMINIQTDNLGNAIYEFTRSALSRIPSPAGLTNFATIICEKDS
Z. rouxii FKKSERYIDLAITLLPSMGVSYANKAMVSLRYNDFTSTIFEAIRGSLIRLPDLNYCTKIYADLILSYD
S. castellii FVKSKEYFDLASLLLPSSGETYLQRGMVYIQTDNLGTAVQEFIKGSLAKSPCPAALSNFKTIILENDS
C. glabrata FQKPLRYLDIASTIVPSVGETFLQRGICYTHTKNFGNAAYQFVRSSLSRLPSDAGIPNFTNLLGDPNG
K. lactis FDDSMRYFMLAKQIVPSVGETYLQEGLVYVQTGNYGHAAYAFMRGSLSRMPTDAGIPNLESIVADASS
A. gossypii NAQAHTYYQHAAQLVPSNGQPYNQLAILAAGKGDQLATVFYYVRSISVKHPFPAAVTNLQMAFSKMID
S. kowalevskii WDCAIRYYKLADTLNPDSGMSHNQLAVIGLADGNHLQATYHLYRALSAREPYPTSNGNLEIEFRKVLA
A. capsulatus WGPAKGYYDLAAEIYPDSGHAPNQLAVIAREDGDHFRCVYHLYRSLASKEPYPQAKANLATEFKRVIA
S. turcica RKQAENFYKQAILVSPTSGHPYNQLALLEASQGDKLSTVFYYIRGIAVKNPFPASTTNLLSTLSSSLD
T. casteneum FRQALRFYFMAHLLFPENGYHKNQMAILYTYQNNDLETLYWLACSSSSFVPMSGAQSNLTKKLSSMQK
S. japonicus ELLAERFYYQALSVAPQIGMPFNQLGTLAGSKYYNVEAMYCYLRCIQSEVSFEGAYGNLKRLYDKAAK
S. pombe TSQAESYYRHAAQLVPSNGQPYNQLAILASSKGDHLTTIFYYCRSIAVKFPFPAASTNLQKALSKALE
YDRALGFYDLAHRTLPGNGMHRNQIAVVWASDECIVESIYWFSSALCSEDPPKSALLNLLKQLIAFYR
(telomere length untested
in fission yeast)
R194G
(short telomeres
in fission yeast)
K252E
LOF:
EST1
est1-K223E
LOF:
EST1
est1-D281K

Figure S4. Additional analysis of the RNA binding domain of Est1

(**A**) Est1 fails to co-IP with Est2 in a strain bearing a mutation in the 5-nucleotide bulge of TLC1 (indicated by the blue box in the cartoon image), which has been previously shown to be required for the Est1-TLC1 interaction (Seto *et al*. 2002) .

Seto, A. G., Livengood, A. J., Tzfati, .Y, Blackburn, E. H., and T. R. Cech, 2002. A bulged stem tethers Est1p to telomerase RNA in budding yeast. Genes Dev 16: 2800–2812.

(**B**) A darker exposure of the image shown in Figure 3A, providing further support for the inability of the C-terminal domain of Est1 to form a complex with TLC1 and Est2.

(**C**) Over-expression of RNA-binding-defective *est1*$^{-}$ mutations in a wild type yeast strain did not confer an ODN phenotype on telomere length, assayed as described in Figure 1. Lanes 1-2, *ADH-EST1*; lanes 3-4, *ADH-est1*-W87E; lanes 5-6, *ADH-est1*-Y136D; lanes 7-8, *ADH-est1*-F98D; lanes 9-10, ADH-*est1*-Y146D. The *est1*-F40E mutation (not included here) failed to show an ODN phenotype in the *yku80*-Δ strain (data not shown).

(**D**) The position of the Est1 RBD identified in this study (indicated by a blue box on the schematic figure of Est1) contradicts a previous report, which relied on a three-hybrid approach to identify a different region of the fission yeast Est1 protein that bound the telomerase RNA (Webb and Zakian 2012. Two of the fission yeast residues implicated in this previously proposed fission yeast RNA binding activity (R194 and K252, highlighted in yellow) are poorly conserved, as shown by an alignment of Est1 proteins from budding and fission yeasts from this region. In addition, mutations in the equivalent residues (K223 and D281) in *S. cerevisiae* resulted in a wild type telomere length (right hand panel), further suggesting that these fission yeast residues do not define a conserved function. A third fission yeast residue (L48) identified in this prior study was in a region of the fission yeast Est1 protein with very limited sequence conservation, which precluded identification of the comparable residue for in vivo analysis in *S. cerevisiae*. In contrast, key residues in the Est1 RBD identified in this study are highly conserved from budding yeast to fission yeast (Fig. 3D).

Webb, C. J. and V. A. Zakian, 2012. *Schizosaccharomyces pombe* Ccq1 and TER1 bind the 14-3-3-like domain of Est1, which promotes and stabilizes telomerase-telomere association. Genes Dev. 26: 82-91.

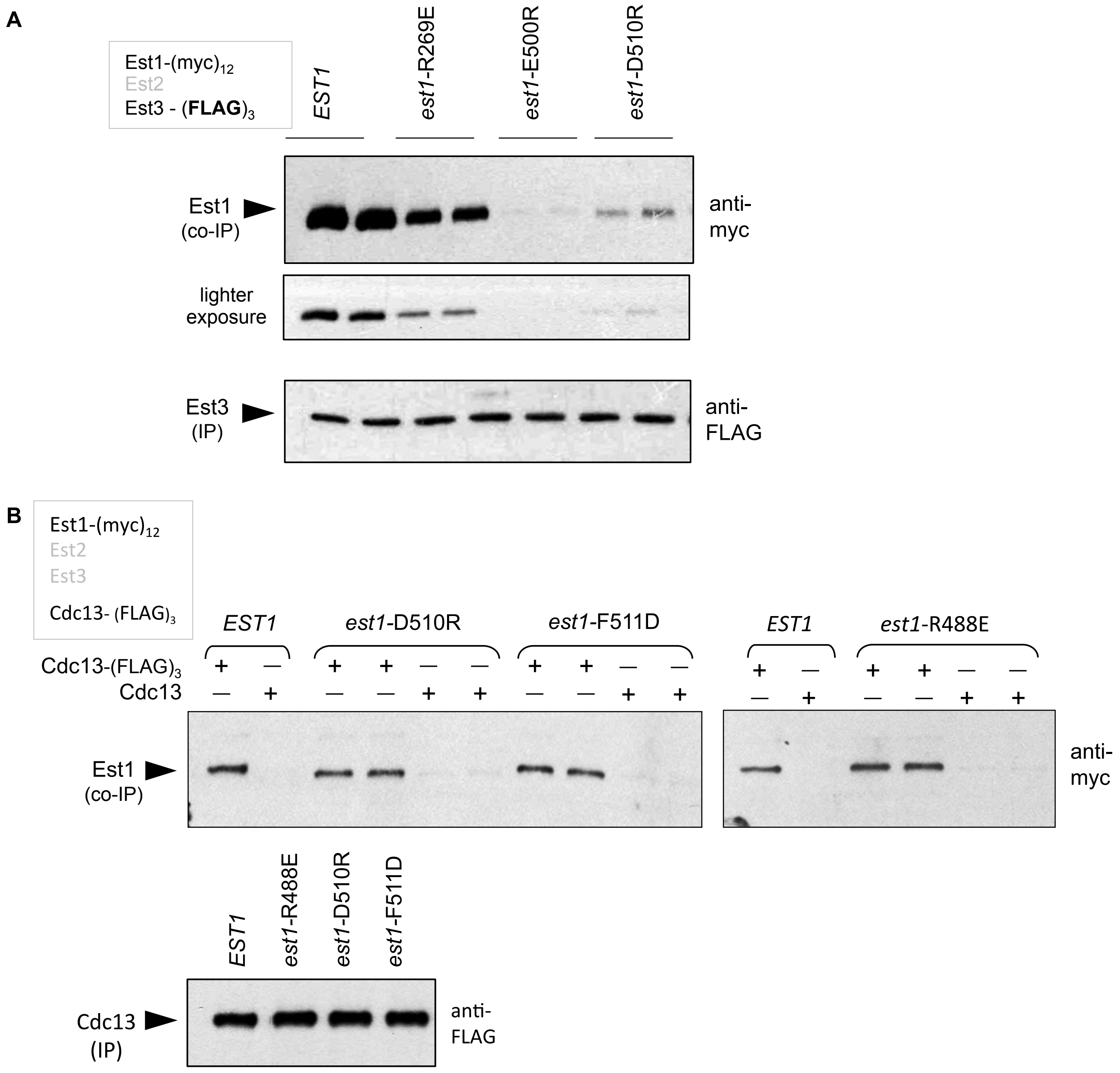

Figure S5. Additional analysis of Est3-binding-defective alleles of *EST1*

(**A**) Comparing the effect of three mutations on the association between Est1 and Est3, tagged with $(myc)_{12}$ and $(FLAG)_3$ epitopes, respectively, following anti-FLAG immunoprecipitation.

(**B**) Association between Est1 and Cdc13 (monitored by anti-FLAG immunoprecipitation with Est1 and Cdc13 tagged with $(myc)_{12}$ and $(FLAG)_3$ epitopes, respectively) was unaffected by *est1*-D510R, *est1*-F111D or *est1*-R488E mutations.

Supplemental Fig. S6
Lubin et al.

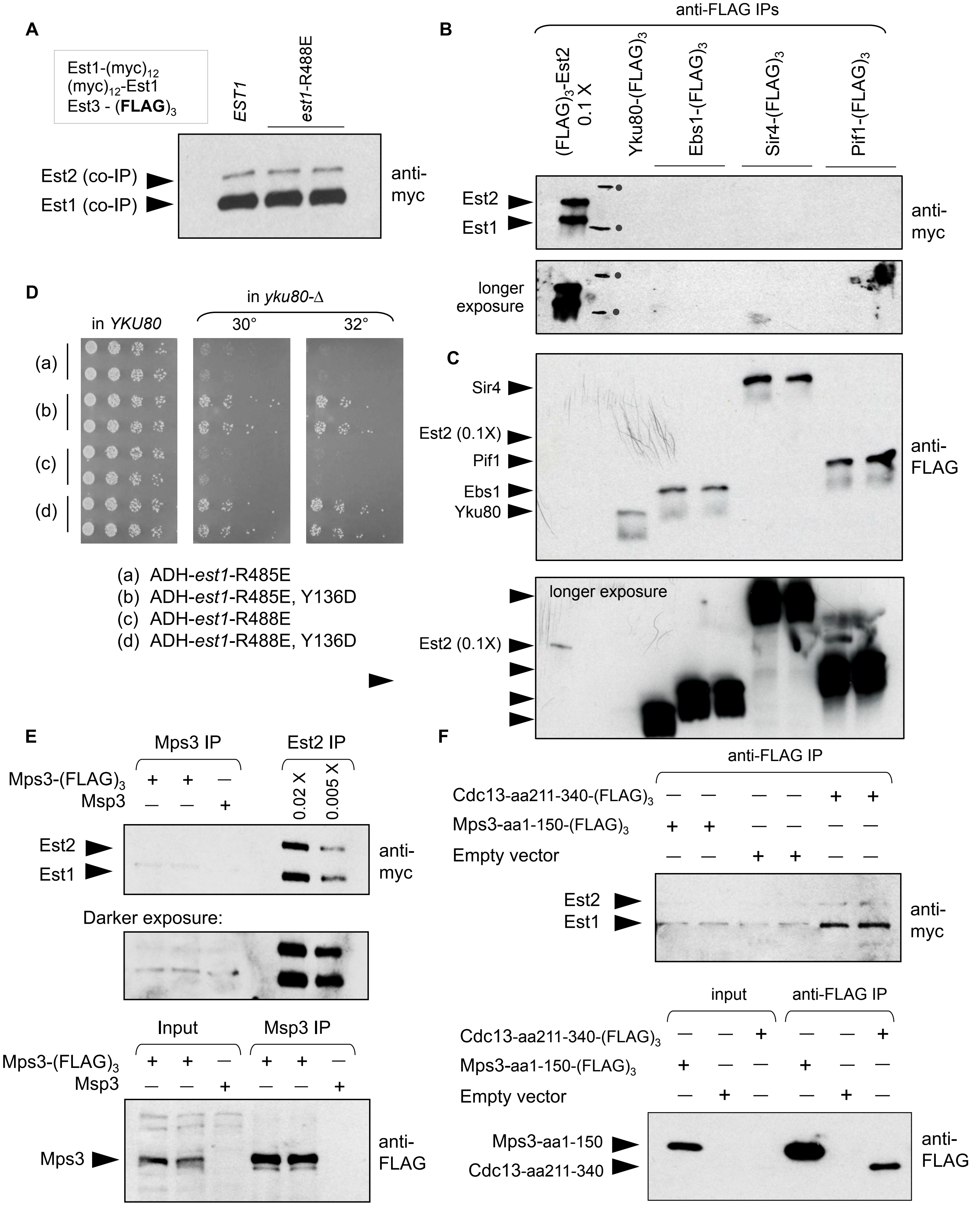

Figure S6. Additional analysis of the fourth function of Est1

(**A**) Anti-FLAG IPs, with the $(FLAG)_3$ epitope on Est3, reveals an altered Est1:Est2 ratio, which we have previously argued is due to a telomerase disassembly complex (Tucey and Lundblad 2014); this ratio is not disrupted in IPs from the *est1*-R488E strain, demonstrating that the disassembly pathway is not altered by this mutation.

Tucey, T. M. and V. Lundblad, 2014 Regulated assembly and disassembly of the yeast telomerase quaternary complex. Genes Dev. 28: 2077-2089.

(**B** and **C**) Anti-FLAG IPs from strains with $(FLAG)_3$ epitopes on Est2, Yku80, Ebs1, Sir4 or Pif1; Est1 and Est2 were tagged with $(myc)_{12}$ in all five strains. The amount of anti-Est2 IP was 0.1X of the amount loaded in the other lanes. (**B**) Est1 association with Est2 was clearly detected in the anti-Est2 IP lane, whereas following anti-FLAG IP of Yku80, Ebs1, Sir4 or Pif1, no association of these four proteins with either Est1 or Est2 could be detected, even in a substantially darker exposure of the anti-myc western. The hand-drawn marks on the anti-myc westerns (indicated by dots) correspond to pre-stained 100 and 150 kDa size markers (visualized on the membrane after transfer). (**C**) Anti-FLAG westerns show that the $(FLAG)_3$-tagged versions of Yku80, Ebs1, Sir4 or Pif1 were readily detectable in the anti-FLAG IPs, relative to the 10-fold reduction Est2 signal.

(**D**) The ODN phenotypes of *est1*-R485E and *est1*-R488E, as assayed by the *yku80*-Δ synthetic lethal assay, were reversed by the presence of the TLC1-binding-defective *est1*-Y136D mutation.

(**E**) A re-examination of the previously proposed interaction between Mps3 and Est1 (Antoniacci *et al.* 2007; Schober *et al.* 2009); anti-FLAG IP were performed as described for part (**C**), above, with a strain containing a $(FLAG)_3$ epitope on Mps3 and $(myc)_{12}$ epitopes on Est1 and Est2. As a control, 0.02X and 0.005X amounts of an anti-FLAG IP from a strain with the $(FLAG)_3$ epitope was on Est2 were included.

Antoniacci, L. M., Kenna, M. A., and R. V. Skibbens, 2007. The nuclear envelope and spindle pole body-associated Mps3 protein bind telomere regulators and function in telomere clustering. Cell Cycle. 6: 75-79.

Schober, H., Ferreira, H., Kalck, V., Gehlen, L. R., and S. M. Gasser, 2009. Yeast telomerase and the SUN domain protein Mps3 anchor telomeres and repress subtelomeric recombination. Genes Dev. 23: 928-938.

Figure S6 (continued). Additional analysis of the fourth function of Est1

(**F**) Because Mps3 is a nuclear envelope integral protein (and thus potentially difficult to assay in immunoprecipitations), we examined the interaction between Est1 and the soluble N-terminal domain of Mps3 (aa 1-150) (Bupp *et al.* 2007), which was tagged with $(FLAG)_3$ and over-expressed by the ADH promoter on a high copy plasmid. Even though the Mps3 N-terminal domain was efficiently expressed, an Est1-Msp3 interaction could not be detected in anti-FLAG IPs. In contrast, association between Est1 and the 130 amino acid recruitment domain of Cdc13 could be readily detected. Based on the results in parts (**D**) and (**E**), we conclude that Mps3 does not exhibit a detectable interaction with either Est1 or Est2.

Bupp, J. M., Martin, A. E., Stensrud, E. S. and S. L. Jaspersen, 2007. Telomere anchoring at the nuclear periphery requires the budding yeast Sad1-UNC-84 domain protein Mps3. J Cell Biol. 179, 845-854.

Table S1. **Yeast strain list.**

Strain	Genotype
YVL2967	*MAT***a** *ura3*-52 *lys2*-801 *trp1*-Δ1 *his3*-Δ200 *leu2*-Δ1
YVL3814	*MAT***a** *est1*-Δ / p *CEN URA3 EST1 ura3*-52 *lys2*-801 *trp1*-Δ1 *his3*-Δ200 *leu2*-Δ1
YVL3142	*MAT***a** *yku80*-Δ / p *CEN URA3 YKU80 ura3*-52 *lys2*-801 *trp1*-Δ1 *his3*-Δ200 *leu2*-Δ1
YVL3528*	*MAT***a** *EST1*-$(Gly)_6$-$(myc)_{12}$ $(FLAG)_3$-$(myc)_{12}$-$(Gly)_6$-*EST2*
YVL3692*	*MAT***a** *est1*-(aa 1-340)-$(Gly)_6$-$(myc)_{12}$ $(FLAG)_3$-$(myc)_{12}$-$(Gly)_6$-*EST2*
YVL3830*	*MAT***a** *est1*-(aa 340-699)-$(Gly)_6$-$(myc)_{12}$ $(FLAG)_3$-$(myc)_{12}$-$(Gly)_6$-*EST2*
YVL4311*	*MAT***a** *est1*-F40R-$(Gly)_6$-$(myc)_{12}$ $(FLAG)_3$-$(myc)_{12}$-$(Gly)_6$-*EST2*
YVL4222*	*MAT***a** *est1*-W87E-$(Gly)_6$-$(myc)_{12}$ $(FLAG)_3$-$(myc)_{12}$-$(Gly)_6$-*EST2*
YVL4132*	*MAT***a** *est1*-F98D-$(Gly)_6$-$(myc)_{12}$ $(FLAG)_3$-$(myc)_{12}$-$(Gly)_6$-*EST2*
YVL3896*	*MAT***a** *est1*-Y136D-$(Gly)_6$-$(myc)_{12}$ *CDC13*-$(FLAG)_3$
YVL3898*	*MAT***a** *est1*-Y146D-$(Gly)_6$-$(myc)_{12}$ *CDC13*-$(FLAG)_3$
YVL3979*	*MAT***a** *EST1*-$(Gly)_6$-$(myc)_{12}$ *CDC13*-$(FLAG)_3$
YVL4553*	*MAT***a** *est1*-W87E-$(Gly)_6$-$(myc)_{12}$ *CDC13*-$(FLAG)_3$
YVL4551*	*MAT***a** *est1*-Y136D-$(Gly)_6$-$(myc)_{12}$ *CDC13*-$(FLAG)_3$
YVL4348*	*MAT***a** *EST1*-$(Gly)_6$-$(myc)_{12}$ *EST3*-$(FLAG)_3$
YVL3813*	*MAT***a** *EST1*-$(Gly)_6$-$(myc)_{12}$ $(myc)_{12}$-$(Gly)_6$-*EST2* *EST3*-$(FLAG)_3$
YVL4025*	*MAT***a** *est1*-Y136D-$(Gly)_6$-$(myc)_{12}$ $(myc)_{12}$-$(Gly)_6$-*EST2* *EST3*-$(FLAG)_3$
YVL3803*	*MAT***a** *EST1*-$(Gly)_6$-$(myc)_{12}$ $(myc)_{12}$-$(Gly)_6$-*EST2*
YVL3903*	*MAT***a** *EST1*-$(Gly)_6$-$(myc)_{12}$ $(myc)_{12}$-$(Gly)_6$-*EST2* *PIF1*-$(FLAG)_3$
YVL3904*	*MAT***a** *EST1*-$(Gly)_6$-$(myc)_{12}$ $(myc)_{12}$-$(Gly)_6$-*EST2* *SIR4*-$(FLAG)_3$
YVL3905*	*MAT***a** *EST1*-$(Gly)_6$-$(myc)_{12}$ $(myc)_{12}$-$(Gly)_6$-*EST2* *EBS1*-$(FLAG)_3$
YVL3906*	*MAT***a** *EST1*-$(Gly)_6$-$(myc)_{12}$ $(myc)_{12}$-$(Gly)_6$-*EST2* *YKU80*-$(FLAG)_3$
YVL4026*	*MAT***a** *EST1*-$(Gly)_6$-$(myc)_{12}$ $(myc)_{12}$-$(Gly)_6$-*EST2* *MPS3*-$(FLAG)_3$
YVL5518*	*MAT***a** *EST1*-$(Gly)_6$-$(myc)_{12}$ *POP1*-$(FLAG)_3$
YVL5529*	*MAT***a** *est1*-Y136D-$(Gly)_6$-$(myc)_{12}$ *POP1*-$(FLAG)_3$
YVL5520*	*MAT***a** *est1*-R485E-$(Gly)_6$-$(myc)_{12}$ *POP1*-$(FLAG)_3$
YVL5519*	*MAT***a** *EST1*-$(Gly)_6$-$(myc)_{12}$ *SME1*-$(FLAG)_3$
YVL5531*	*MAT***a** *est1*-Y136D-$(Gly)_6$-$(myc)_{12}$ *POP1*-$(FLAG)_3$
YVL5521*	*MAT***a** *est1*-R485E-$(Gly)_6$-$(myc)_{12}$ *POP1*-$(FLAG)_3$

* *leu2 trp1 ura3-52 prb1*¯ *prc1*¯ *pep4-3 bar1*-Δ::KAN

Table S1 (continued) Yeast strain list.

wild type parent:	YVL3528	YVL3979	YVL4348
est1-K444E	YVL4745	YVL4301	n.a.
est1-R447E	YVL4747	YVL4340	n.a.
est1-R485E	YVL4396	YVL4303	YVL4382
est1-R488E	YVL4398	YVL4325	YVL4384
est1-E500R	YVL4749	YVL4305	YVL5039
est1-D510R	YVL4402	YVL4307	YVL4974
est1-F511D	YVL4404	YVL4344	YVL4975
est1-R269E	YVL4394	n.a.	YVL4555
est1-K555E	YVL5480	YVL4309	n.a.
est1-K559E, W566D	YVL5477	YVL4342	n.a.

Table S2. Plasmid list.

Plasmid	Description	Vector backbone
pVL367	*CEN URA3 EST1*	YCplac 33
pVL809	2 μ *HIS3 ADH-EST1*	pRS423
pVL5187	*URA3 EST1*-$(Gly)_6$-$(myc)_{12}$	YIplac211
pVL6685	2μ *TRP1 ADH-CDC13*-aa211-340-$(FLAG)_3$	YEplac112
pVL6732	2μ *TRP1 ADH-MPS3*-aa1-150-$(FLAG)_3$	YEplac112

wild type parent:	pVL367	pVL809	pVL5187
est1-F40E	pVL7343	pVL7759	pVL6889
est1-W87E	pVL6793	pVL7399	pVL6636
est1-F98D	pVL6794	pVL4681	pVL6071
est1-Y136D	pVL6803	pVL7356	pVL6072
est1-Y146D	pVL6804	pVL4688	pVL6073
est1-K444E	pVL6478	pVL4534	pVL5236
est1-R447E	pVL6479	pVL4512	pVL6686
est1-R485E	pVL6481	pVL4517	pVL6687
est1-R488E	pVL6482	pVL4519	pVL6688
est1-E500R	pVL6485	pVL4826	pVL6689
est1-D510R	pVL6486	pVL4525	pVL6691
est1-F511D	pVL6487	pVL6473	pVL6690
est1-R269E	pVL6820	pVL4607	pVL6882
est1-K555E	pVL6490	pVL4612	pVL6692
est1-K559E, W566D	pVL7761	pVL4695	pVL6807
